# Identifying Key Questions and Challenges in Microchimerism Biology

**DOI:** 10.1002/advs.202514969

**Published:** 2025-10-24

**Authors:** Kristine J. Chua, Rachel C. Quilang, Katja Sallinger, Christina Athena Aktipis, Petra Arck, Diana W. Bianchi, Hyun‐Dong Chang, Henderson J. Cleaves, Michael Eikmans, Heidi E. S. Fjeldstad, David Haig, Whitney E. Harrington, William Horsnell, Daniel P. Jacobsen, Mads Kamper‐Jørgensen, Sami B. Kanaan, Kiarash Khosrotehrani, Nathalie C. Lambert, J. Lee Nelson, Maria B. Olsen, Tiffany D. Pan, Jelmer R. Prins, Frank A. Schildberg, Anne Cathrine Staff, Anders Ståhlberg, Ina A. Stelzer, Christopher Urbschat, Sing Sing Way, Melissa A. Wilson, Jody Ye, Thomas Kroneis, Amy M. Boddy

**Affiliations:** ^1^ Department of Anthropology University of Notre Dame Notre Dame 46556 USA; ^2^ Department of Immunology Leiden University Medical Center Leiden 2333 ZA Netherlands; ^3^ Department of Cell Biology Histology & Embryology Gottfried Schatz Research Center Medical University of Graz Graz 8010 Austria; ^4^ Center for Evolution and Medicine Arizona State University Tempe 85287 USA; ^5^ Department of Psychology Arizona State University Tempe 85287 USA; ^6^ Division of Feto‐Maternal Medicine Department of Obstetrics and Fetal Medicine University Medical Center Hamburg‐Eppendorf 20246 Hamburg Germany; ^7^ Hamburg Center for Translational Immunology University Medical Center Hamburg‐Eppendorf 20246 Hamburg Germany; ^8^ National Human Genome Research Institute National Institutes of Health Bethesda 20892 USA; ^9^ National Institute of Child Health and Human Development National Institutes of Health Bethesda 20892 USA; ^10^ German Rheumatology Research Center A Leibniz Institute (DRFZ) 10117 Berlin Germany; ^11^ Technische Universität Berlin Department for Cytometry Institute of Biotechnology 13355 Berlin Germany; ^12^ Department of Chemistry Howard University Washington D.C. 20059 USA; ^13^ Division of Obstetrics and Gynaecology Oslo University Hospital Oslo 0424 Norway; ^14^ Department of Organismic and Evolutionary Biology Harvard University Cambridge 02138 USA; ^15^ Department of Pediatrics University of Washington School of Medicine Seattle 98195 USA; ^16^ Center for Global Infectious Disease Research Seattle Children's Research Institute Seattle 98105 USA; ^17^ MRC Centre for Medical Mycology University of Exeter Exeter EX4 4QJ UK; ^18^ Institute for Infectious Disease and Molecular Medicine Centre for Infectious Disease Research in Africa and Division of Immunology University of Cape Town Cape Town 7701 South Africa; ^19^ Faculty of Medicine University of Oslo Oslo 0316 Norway; ^20^ Department of Public Health University of Copenhagen Copenhagen DK‐1165 Denmark; ^21^ Translational Science and Therapeutics Division Fred Hutchinson Cancer Center Seattle 98109 USA; ^22^ Frazer institute The University of Queensland Woolloongabba 4072 Australia; ^23^ Translational Research institute The University of Queensland Woolloongabba 4072 Australia; ^24^ INSERM UMRs 1097 ARTHEMIS Aix Marseille University Marseille 13284 France; ^25^ Medicine, Rheumatology University of Washington and Fred Hutchinson Cancer Center Seattle 98109 USA; ^26^ Research Institute of Internal Medicine Oslo University Hospital Oslo 0424 Norway; ^27^ Center for Studies in Demography and Ecology University of Washington Seattle 98195 USA; ^28^ Obstetrics and Gynecology University Medical Center Groningen University of Groningen Groningen 9700 AB The Netherlands; ^29^ Department of Orthopedics and Trauma Surgery University Hospital Bonn 53127 Bonn Germany; ^30^ Sahlgrenska Center for Cancer Research Department of Laboratory Medicine Wallenberg Centre for Molecular and Translational Medicine Science for Life Laboratory Institute of Biomedicine Sahlgrenska Academy University of Gothenburg Gothenburg 405 30 Sweden; ^31^ Department of Clinical Genetics and Genomics Sahlgrenska University Hospital Region Västra Götaland Gothenburg SE 413 45 Sweden; ^32^ Pathology Center for Perinatal Discovery University of California San Diego La Jolla 92037 USA; ^33^ Center for Inflammation and Tolerance Cincinnati Children's Hospital Cincinnati 45229 USA; ^34^ University of Cincinnati College of Medicine Cincinnati 45219 USA; ^35^ National Human Genome Research Institute National Institutes of Health Bethesda Bethesda 20892 USA; ^36^ Division of Endocrinology Department of Medicine Albert Einstein College of Medicine Bronx 10461 USA; ^37^ Department of Anthropology University of California Santa Barbara Santa Barbara 93106 USA

**Keywords:** autoimmunity, detection methods, evolution, immune tolerance, maternal‐fetal interaction, microchimerism, pregnancy

## Abstract

Microchimerism research has recently gained renewed attention despite known existence of these rare cells for decades. Fetal and maternal microchimeric‐derived cells may have functional capabilities, and are increasingly associated with both beneficial and adverse health outcomes. Yet, establishing the role of microchimerism in health has been largely constrained methodologically and theoretically. The Microchimerism, Human Health, and Evolution Project address these challenges by calling on 29 leading microchimerism experts to put forth key research questions that can substantially advance the field. Seven major categories are identified: function and mechanism; microchimerism in interventions, treatment, and transplant; mapping “generational microchimerism”; evolution; microchimerism detection; appropriate experimental model systems; and definition of microchimerism. Identifying these questions ‐ and continuing to find answers ‐ will be crucial for advancing the knowledge of microchimerism in health and disease.

## Graphical abstract

This study identifies key unanswered questions about microchimerism, the presence of cells from one individual living in another. Experts highlight how these cells may affect health, pregnancy, and disease. This study outlines research priorities and challenges in detecting and studying these rare microchimeric cells, aiming to guide future discoveries in biology and medicine.

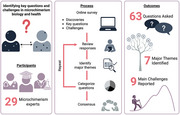

## 1 Introduction

Stories from Greek mythology depicted a fantastical fire‐breathing, three‐headed creature consisting of distinct lion, snake, and goat features. It was called the Chimera, for being composed of multiple animal parts and symbolizing chaos and destruction (derived from the Greek for a she‐goat, *khímaira*).

The term “chimerism” is now used in biology to refer to an organism that is composed of cells from two or more zygotes. Chimerism gained prominence in the fields of immunology and transplantation during the mid‐20th century to describe the presence of at least two genetically distinct cell populations within a single individual.^[^ ^1^ ^]^ Although the formal study of microchimerism dates back to 1893 when Georg Schmorl first reported thrombi containing placental multinucleated syncytial giant cells in the lungs of women who had died of eclampsia,^[^ ^2^, ^3^ ^]^ the term “microchimerism” was not used until the 1970s by Liégeois et al.^[^ ^4^ ^]^ (*4*) to broadly refer to the phenomenon of organisms harboring a small (or “micro”) number of genetically distinct (i.e., allogeneic) cells.

Despite increasing descriptions of microchimerism in a variety of physiological contexts, many questions still remain. It is now known that microchimerism exists across many different mammalian species, suggesting it arose at least 100 million years ago with the origin of eutherian mammals.^[^ ^5^ ^]^ Whether the same or similar microchimerism phenomena occur in deeper branches of animalia remains uncertain. The ability to tolerate genetically and antigenically distinct cells within a single organism likely requires fundamental immune adaptations. These adaptations likely evolved early in multicellular life. However, internal gestation in placental mammals brings maternal and fetal tissues into close contact, potentially increasing bidirectional cellular exchange.

**Figure 1:**
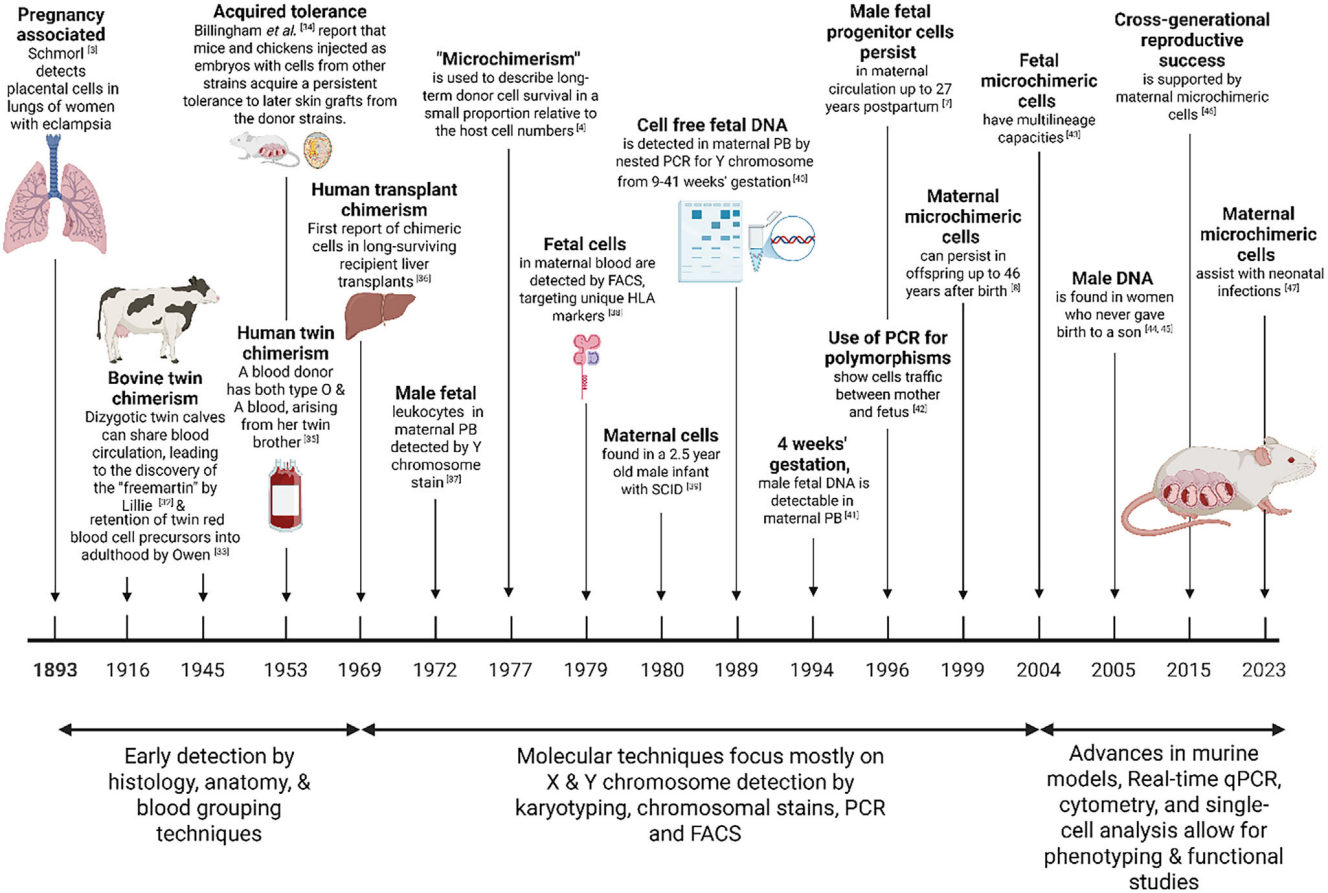
Major historical discoveries contributing to the field of microchimerism. Many key findings in the areas of immunology and transplantation have led to the emergence of the microchimerism field. Advancements in technology have enabled greater characterization and understanding of the functional roles of microchimeric cells. PB, peripheral blood; FACS, fluorescence‐activated cell sorting; HLA, human leukocyte antigen; SCID, severe combined immunodeficiency; PCR, polymerase chain reaction; qPCR, quantitative polymerase chain reaction. Image created with Biorender.com.

We also know now that microchimerism is multi‐directional in mammals. Fetal microchimerism refers to cell transfer from the fetus to mother. Maternal microchimerism describes maternal cells that are transferred to the offspring. Microchimerism can also arise from twinning, as well as organ and stem cell transplantation.^[^ ^6^ ^]^ There is evidence that microchimeric cells can persist for decades, potentially even for entire organismal lifetimes.^[^ ^7^, ^8^ ^]^ Both fetal‐ and maternal‐derived microchimeric cells have been found to differentiate into almost any cell type and colonize almost any tissue, including crossing the blood‐brain barrier to become neurons.^[^ ^9^, ^10^, ^11^ ^]^ Fetal microchimerism has been proposed to play a role in maternal wound healing.^[^ ^12^, ^13^ ^]^ Yet, fetal microchimerism has also been associated with pregnancy complications, such as pre‐eclampsia, spontaneous abortion, and placental dysfunction,^[^ ^14^, ^15^, ^16^, ^17^, ^18^, ^19^, ^20^ ^]^ cancer,^[^ ^21^, ^22^, ^23^ ^]^ and autoimmune diseases.^[^ ^24^, ^25^, ^26^ ^]^ Similarly, maternal microchimerism has been proposed to play a role in promoting offspring immune development,^[^ ^27^ ^]^ such as susceptibility to infection,^[^ ^28^ ^]^ vaccine response,^[^ ^29^ ^]^ and propensity for autoimmune disorders.^[^ ^30^, ^31^ ^]^ Immunosuppression by T‐regulatory cells is thought to be a main mechanism driving host tolerance associated with persistence of microchimeric cells.^[^ ^31^ ^]^ See **Figure** **1** and Table S1 (Supporting Information) for a detailed historical overview summarizing the major discoveries in microchimerism research to date.

A multidisciplinary community can overcome the methodological and theoretical challenges that have limited understanding what microchimeric cells do and how they work. Microchimeric cells are rare (< 1%, and often in the order of 1 in 10^6^ host cells) making them difficult to identify and characterize. With the advancement of rare cell detection methods, the field has experienced a major revitalization allowing for old and new questions to be addressed. This led to the launching of research consortia, such as the Microchimerism, Human Health and Evolution Project in 2021 (https://www.microchimerism.info/) and Collaborative Research Center 1713 in 2025 (https://www.crc1713.org). Our international teams include cellular and reproductive biologists, immunologists, anthropologists, evolutionary theorists, immunogeneticists, and clinicians. We integrate technologies from transplantation research, mouse models, immunohistochemistry, and single‐cell sequencing alongside an evolutionary perspective to explore the reason for, and consequences of, the presence and persistence of microchimerism.

We herein explore the state of the field of human microchimerism research and attempt to establish key concepts, questions, and approaches for future work. Identifying these questions will provide a cornerstone for future microchimerism research as it relates to human health and evolutionary biology.

## 2 Methods

To identify key research questions in microchimerism biology and health we followed a protocol previously used by Sutherland et al.^[^ ^48^ ^]^ and adopted by Dujon et al.^[^ ^49^ ^]^ We identified 29 leading experts based on their contributions to the microchimerism field. As part of a snowball recruitment strategy, we asked participants to provide additional experts, who were all contacted following the initial wave of the study. Those interested filled out an online survey indicating: 1) pivotal discoveries since the inception of the field of microchimerism research; 2) between 3–5 key questions that could advance the field (total number of questions, *Q* = 91); and 3) the main challenges the field faces. To analyze the responses, we collapsed similar and repeated questions into single questions and split multi‐faceted questions, resulting in a total number of 63 unique questions. Questions were edited lightly to ensure clarity. Responses were accompanied by a 300‐word explanation detailing why the questions provided were of key importance, their impact on the field, why these questions have remained unanswered, along with associated references. See Table S2 (Supporting Information) for the full survey.

We identified seven major themes (**Table** **1**) among the questions submitted by each co‐author. We note that the themes under which these questions fall are not mutually exclusive and will naturally overlap; content guided how these questions were categorized. All co‐authors were consulted for the validation of the finalized themes. Additional themes were identified using the aforementioned methods to describe the challenges faced when attempting to answer these key questions. Explanations provided by the experts and ongoing discussions were used to define and justify each theme. See Table S3 (Supporting Information) for the full list and categorizations of the questions, respectively, as well as Table S4 (Supporting Information) associated challenges.

**Table 1:** Main Themes and Definitions. Q = 72 after combining questions that were similar and separating questions that had several topics. Frequency of themes raised by authors is shown.

Theme	Definition	Frequency (of 100 %) of themes raised by authors
Function and mechanism	Fundamental mechanisms and functions of microchimerism as it pertains to disease vulnerability or protection (e.g., autoimmunity, cancer, infection), maternal and fetal immune tolerance, cellular trafficking	59%
Microchimerism in interventions, treatment, and transplantation	Practical applications of microchimerism research in clinical settings, including therapeutic interventions, treatments, and transplantation	10%
Evolution	Evolutionary perspectives for generating predictions on the role of microchimerism and its existence in humans and other species	10%
Mapping “generational microchimerism”	Microchimerism trafficking within and between generations	10%
Microchimerism detection	Development and refinement of techniques for detecting microchimerism	8%
Definition of microchimerism	Establishment of a precise definition and scope of microchimerism	3%
Appropriate experimental model systems	Development and validation of suitable experimental models to study microchimerism	2%

## 3 Results

### 3.1 Theme: Function and Mechanism

The theme, “Mechanism and Function”, emerged as the largest percentage of questions asked among leading experts in the field of microchimerism (**Table** **2**). Due to the significant interest in this area, we introduced sub‐themes to provide a more nuanced exploration of this theme. These sub‐themes include cell interaction, cell transmission, disease vulnerability or protection, persistence of microchimerism, plasticity, quantity of microchimerism, and tissue dependent microchimerism trafficking.

**Table 2:** Frequency of Main Themes and Subthemes. Q = 63 after combining questions that were similar and separating questions that had several topics. Frequency of themes raised by authors is shown.

Theme	Subtheme 1	Subtheme 2	Frequency (of 100 %) of themes raised by authors
**Appropriate experimental model systems**			2%
**Definition of microchimerism**			3%
**Evolution**			10%
	Adaptation		6%
	Comparative approach		3%
**Function and mechanism**			59%
	Disease vulnerability or protection		30%
	Maternal outcomes	Fetal sex and maternal health	2%
		Maternal health and immunity	3%
		Maternal mental health	2%
		Microchimerism in disease	11%
	Offspring outcomes	Developmental origins of health and disease	6%
		Fetal health and immunity	5%
		Pregnancy outcomes	2%
	Cell interaction		2%
	Cell transmission		5%
	Persistence of microchimerism Cell transmission		5%
	Plasticity		3%
	Quantity of microchimerism		8%
	Tissue‐dependent microchimerism trafficking		6%
**Microchimerism detection**			8%
**Microchimerism in interventions, treatment, and transplantation**			10%
**Mapping "generational microchimerism"**			10%
	Intergenerational effect		3%
	Transgenerational effect		6%

#### 3.1.1 Subtheme: Disease Vulnerability or Protection

Disease vulnerability or protection was the most extensively discussed sub‐theme (Table 2). This includes important areas concentrating on maternal outcomes such as developmental origins of health and disease; microchimerism in disease; maternal health and immunity; and maternal mental health. Other areas focusing on fetal outcomes include fetal sex and maternal health; fetal health and immunity, as well as pregnancy outcomes. Within the disease vulnerability or protection sub‐theme, questions focused on early life exposure to maternal environmental cues and disease outcomes. These extend to infectious diseases even if not explicitly discussed. For ease of readability, this section has been further broken down by these categories.

**Figure 2:**
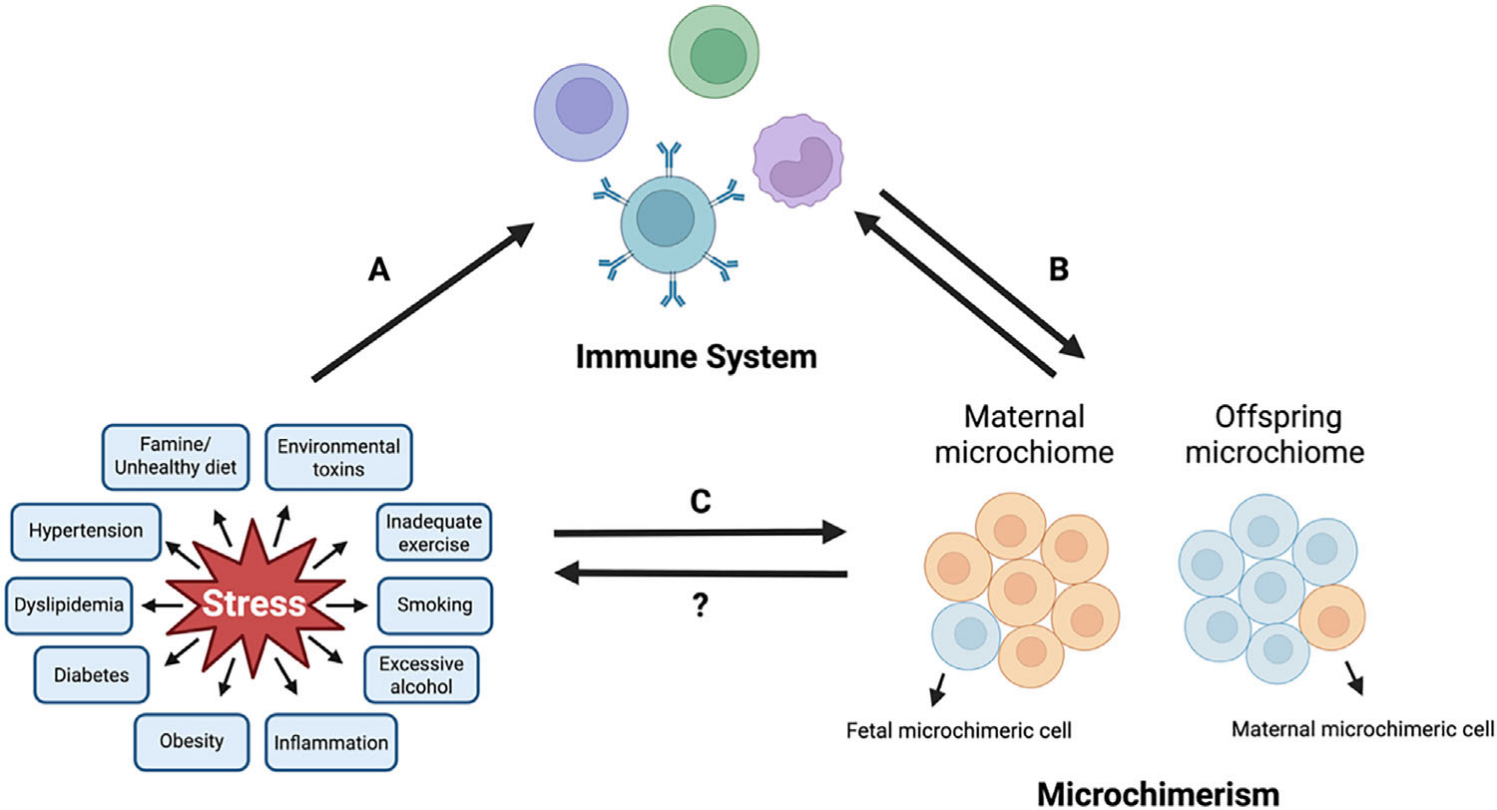
Conceptual model. While not an exhaustive list, chronic stress encompasses maternal psychosocial and physical stress, nutrition, and trauma, as well as some of its associated diseases that have been important in understanding DOHaD. The immune system is represented by general immune cells. The maternal and offspring microchiome are representations of the diversity of cells within mother‐fetal dyads. Maternal microchimerism is represented as the small orange cell among the blue fetal cells. Fetal microchimerism is represented as the small blue cell among the orange maternal cells. A) Relationship between chronic stress and immune system. B) Relationship between immune system with maternal and fetal microchimerism. C) Unknown relationship between chronic stress with maternal and fetal microchimerism. Image created with Biorender.com.

The first main category of the disease vulnerability sub‐theme relates to the developmental origins of health and disease (DOHaD), which has become a dominant framework to study intergenerational health.^[^ ^50^, ^51^ ^]^ This is particularly noticeable in the context of stress (reviewed in refs. [52, 53]) and intergenerational trauma.^[^ ^54^, ^55^, ^56^ ^]^ That is, mothers may experience psychosocial and physiological stimuli that impacts their biology in various ways. These biochemical cues are then transmitted to the fetus, which has different periods of specific organ vulnerability across the gestational development. DOHaD brings attention to how social determinants of health shape our biology, which cannot be ignored in microchimerism research. Thus, one fruitful area of research can investigate how chronic stress, immune regulation, and the contributions of maternal and fetal microchimerism impact pregnancy (**Figure** **2**). Relatedly, there is a Eurocentric bias where our understanding of microchimerism is mostly derived from cohorts with European ancestry, with relatively fewer studies focus on individuals from Africa (e.g., South Africa;^[^ ^29^, ^57^ ^]^ Tanzania;^[^ ^28^ ^]^ Uganda^[^ ^58^ ^]^), and East Asia (e.g., China;^[^ ^59^ ^]^ Japan^[^ ^60^ ^]^). Future research should examine whether microchimerism varies across populations in frequency, distribution, or health impact due to ecological, social, and genetic diversity factors.

Related questions posed by the experts

**MATERNAL OUTCOMES**:

**Maternal mental health**

- Do microchimeric cells in the maternal brain, and interactions with the immune system, especially in the immediate post‐partum time, contribute to challenges in maternal postpartum mental health?

**Fetal sex and maternal health**

- Does fetal origin microchimerism originating from male and female fetuses similarly impact maternal health later in life?

**OFFSPRING OUTCOMES**:

**Developmental origins of health and disease**

- Can understanding the impact of microchimerism on the maternal and offspring immune systems and vice versa help with immune‐related disorders, including but not limited to autoimmune disease, infectious disease susceptibility, fertility issues and even transplantation research?
- Are the associations between microchimerism and later‐in‐life diseases a reflection of microchimerism serving a functional biological role, or is microchimerism simply a byproduct of pregnancy?
- What are the determinants of maternal microchimerism transfer in utero and *post‐natally* ‐ specifically, what is the relationship between the maternal immune system and the cells that are transferred?
- What is the role of microchimerism in development?

The second main category of the disease vulnerability sub‐theme relates to disease outcomes. Although microchimerism is common in healthy individuals, the potential impact of microchimerism on the health of parous and/or gravid women and their children has only recently been appreciated, as both maternal and fetal microchimerism have been implicated in various autoimmune and non‐autoimmune diseases. These diseases are briefly reviewed, but not limited to those listed in **Table** **3**. Interpreting function and disease vulnerability within microchimerism research has limitations, as current findings depend heavily on methodological choices. Understanding disease risk is particularly complex, as results can vary significantly based on whether microchimerism is measured in blood or tissue, for example in cancer (reviewed in^[^ ^61^ ^]^). Future research must establish how microchimerism could serve as a reliable biomarker, guiding its potential clinical applications.

**Table 3:** Overview of fetal, maternal, and male origin microchimerism implicated in human autoimmune and non‐autoimmune diseases. Microchimerism and its the role in human autoimmune and non‐autoimmune diseases remain unclear. The “hypothesized role” column is based on author interpretations of reported findings: “increased risk” suggests microchimerism has been proposed to contribute to disease, whereas “decreased risk” suggests it may play a protective role. “Associated” reflects the possibility of multifactorial causes. An asterisk (*) indicates diseases where microchimerism shows dual effects, either increasing risk or providing protection, depending on the context. Diseases were searched using the Microchimerism Literature Atlas (https://literature‐atlas.microchimerism.info/).

Disease implication	Type of microchimerism	Hypothesized role	References
Asthma	Maternal microchimerism	Protective	[68]
Autoimmune thyroid disease (AITD)	Fetal microchimerism	Increased risk	[69]
Biliary atresia	Maternal microchimerism	Increased risk	[70]
Brain function	Maternal microchimerism	Protective	[71]
Breast cancer	Fetal microchimerism	Reduced risk/protective	[72, 73]
Cervical cancer	Fetal microchimerism	Increased risk	[21]
Colon cancer	Fetal microchimerism	Increased risk	[73]
COVID‐19	Fetal microchimerism	Protective	[74]
Hepatitis C infected liver	Fetal microchimerism	Tissue repair	[75]
Infectious disease tolerance	Maternal microchimerism	Protective	[27, 47]
Juvenile dermatomyositis	Maternal microchimerism	Increased risk	[76, 77]
Melanoma	Fetal microchimerism	Increased risk	[78]
Multiple sclerosis (MS)	Fetal microchimerism Maternal microchimerism	Increased risk Increased risk	^[^ ^79^, ^80^ ^]^
Neonatal lupus syndrome	Maternal microchimerism	Increased risk and/or contribute to tissue repair	[82]
Ovarian cancer	Male origin microchimerism	Reduced risk	[83]
Plasmodium falciparum	Maternal microchimerism	Protective	[28]
Polymorphic eruptions of pregnancy	Fetal microchimerism	Increased risk	[84]
Preeclampsia	Fetal microchimerism	Increased risk	[85, 86]
Primary Biliary Cirrhosis	Fetal microchimerism	Present with no significant role	[87, 88, 89]
Rheumatoid arthritis	Fetal microchimerism	Increased risk/ Protective*	^[^ ^90^, ^91^, ^92^ ^]^ ^[^ ^93^, ^94^ ^]^*
Severe combined immunodeficiency	Maternal microchimerism	Protective	[39]
Sjögren's syndrome	Fetal microchimerism	Increased risk	[95]
Systemic lupus erythematosus	Fetal microchimerism	Increased risk	[96]
Systemic sclerosis (scleroderma)	Fetal microchimerism Maternal microchimerism	Increased risk/associated Associated	^[^ ^97^, ^98^, ^99^, ^100^, ^101^ ^]^
Type 1 diabetes	Maternal microchimerism	Tissue repair	[102, 103, 104]
Uterine cancer	Fetal microchimerism	Reduced risk	[105]
Viral respiratory tract infections	Maternal microchimerism	Protective	[27]

Due to the association between certain autoimmune diseases and pregnancy, as well as increased incidence in women, many studies have focused on the potential role of microchimerism in autoimmune disease.^[^ ^62^ ^]^ Just as some (human leukocyte antigen) HLA genes are associated with the risk of autoimmune disease, others are associated with protection. For example, women with rheumatoid arthritis (RA) often have remissions or improvement of RA during pregnancy,^[^ ^63^ ^]^ which is most often due to maternal‐fetal HLA disparity.^[^ ^64^ ^]^ The DERAA sequence, a five amino acid motif encoded by *HLA‐DRB1* alleles is considered RA‐protective, however RA risk is increased in DERAA^‐^ women with DERAA^+^ children prior to onset. As shown by Kanaan et al.,^[^ ^26^ ^]^ detection of DERAA^+^ microchimeric pregnancy‐derived cells in the blood of these women may act as triggers to stimulate an autoimmune reaction by activation of naturally occurring DERAA‐directed T cells in DERAA^‐^ individuals.^[^ ^65^ ^]^ While microchimerism has been associated with a number of different autoimmune diseases,^[^ ^31^, ^66^ ^]^ we are only beginning to understand the mechanisms by which microchimerism may contribute to disease vulnerability or protection. As technology develops, there is the opportunity to expand into lesser investigated areas, such as cardiovascular disease and infectious diseases that the maternal immune system is exposed to, which may assist in neonatal immunity against early life infections.^[^ ^27^, ^28^, ^47^, ^67^ ^]^

Related questions posed by the experts

**MATERNAL OUTCOMES**:

**Mic rochimerism in dise ase**

- What is the role of microchimerism in cardiovascular disease, (CVD), particularly atherosclerosis and CVD phenotypes specific to women?
- How does microchimerism contribute to autoimmune diseases, especially regarding the balance between tolerance and autoimmunity?
- What is the contribution of microchimerism to cancer development?
- What is the role of microchimerism in the process of aging?
- Can fetal cells cause or “cure” disease?
- What types of maternal injuries or diseases recruit fetal cells?
- What is the role of endometrial/uterine microchimeric cells in fertility, decidualization, implantation, placental health, pregnancy health, and subsequent pregnancies?

**Maternal health and immunity**

- What is the functional role of fetal microchimerism during pregnancy, including impacts on maternal outcomes and immune tolerance?
- Does maternal microchimerism shape fetal immune cells toward tolerance only? Or can maternal cells educate fetal T cells toward autoimmunity? Can maternal autoimmunity be transferred to the offspring?

**OFFSPRING OUTCOMES**:

**Fetal health and immunity**

- How does maternal microchimerism support fetal and neonatal development and immunity?
- What is the functional role of maternal microchimerism in infant immunity, both via direct antigen‐specific T / B cells or by “educating” the fetal and infant response? Can transferred lymphocytes exert an effect on offspring immunity to infection independently of antigen experience)?
- Are antigen‐specific B cells subject to maternal microchimerism and do they contribute to our antibody repertoire?

**Pregnancy outcomes**

- Does fetal microchimerism play a role in immune tolerance of the fetus during pregnancy? If yes, what is the mechanism?

#### 3.1.2 Subthemes: Cell Transmission and Tissue‐Dependent Trafficking

Cell transmission and tissue‐dependent trafficking refers to the mechanism by which microchimeric cells are transferred from donor to recipient.

The mechanisms involved in cell transfer across the human placental barrier and fetal membranes remain poorly investigated. It is unknown if microchimeric cells are actively recruited or passively transported. However, one study^[^ ^106^ ^]^ used a human lineage mesenchymal stromal cell line transferred to a pregnant rat to show that these cells trafficked using VEGF‐A and integrin‐dependent pathways across the hemochorial placenta to fetal tissues, suggesting active transport for maternal microchimerism. In addition, the composition of maternal microchimerism does not reflect maternal peripheral blood, suggesting selective transfer (e.g., more T cells than their frequency in maternal PBMC).^[^ ^107^ ^]^ Regardless, naturally acquired microchimerism is considered asymmetrical, with more fetal microchimerism being transferred to the mother than maternal microchimerism to the fetus.^[^ ^108^ ^]^ The exact time point at which migration of fetal cells to the mother and vice versa occurs is not known. However, cell trafficking is more likely to occur once maternal spiral arteries have been remodeled establishing blood circulation.^[^ ^109^ ^]^ Migration may occur even earlier due to uterine histiotrophic nutrition,^[^ ^110^ ^]^ which could also provide passage for microchimeric cells even before the blood circulation is fully established, as fetal Y chromosome DNA, suggestive of cells of fetal origin, has been detected in the maternal circulation as early as seven weeks of gestation.^[^ ^111^ ^]^

It is important to note that during pregnancy, human uterine spiral arteries are remodeled into larger conduit vessels of low blood pressure with high vascular conductance opening toward the intervillous space with little or no impedance to blood flow.^[^ ^112^ ^]^ In this low speed and low pressure environment, it is likely that maternal microchimeric cells actively traffic across the placental barrier. It is suspected that the mechanism of cell migration may include the steps of a diapedesis‐like process: cell capture, cell tethering/rolling, cell adhesion and transmigration mitigated by adhesion molecules and their cognate ligands.^[^ ^113^ ^]^ The existence of a controlled mechanism (i.e., active recruitment) for cell trafficking at the placental barrier is supported by the expression of molecules relevant to the diapedesis process in murine and human placentas.^[^ ^114^, ^115^ ^]^ To study this phenomenon, reporter murine models and ex vivo human placental perfusion models have been used to examine maternal to fetal, and fetal to maternal trafficking.^[^ ^113^ ^]^ However, it cannot be ruled out that maternal cells may also transfer passively to placental vessels by the mechanical trapping of cells in a narrow intervillous space among the microvilli border of the syncytiotrophoblast layer,^[^ ^113^, ^116^ ^]^ or during fetal surgical intervention.^[^ ^117^ ^]^ Alternatively, maternal cells may enter the placenta and cord blood during delivery, though this possibility requires further investigation.

Considering that the pressure within fetal villi vessels of the placenta is greater than within the intervillous space, which protects fetal vessels against collapse,^[^ ^118^ ^]^ it is suspected that there is a component of passive leakage of fetal cells into the maternal circulation. Passive leakage of fetal cells across compromised placental barriers, during fetal‐maternal hemorrhage, or during fetal surgical intervention^[^ ^117^ ^]^ is also possible. This was seen by Bianchi et al.^[^ ^119^ ^]^ where significant fetal‐maternal transfusion of cells, even after a first‐trimester elective termination procedure was observed. Peterson et al.^[^ ^19^ ^]^ also documented fetal microchimeric cell transfer in maternal peripheral blood from both miscarriage and elective termination, with surgical termination resulting in higher levels of fetal microchimeric cells compared to medical terminations. These studies imply that a component of passive cell transmigration does occur, although the specific pathway and timing is unclear. Furthermore, the phenotype and amounts of fetal cells acquired during these events could significantly affect our understanding of fertility as well as its long‐term implications. This is important as approximately 10 ‐ 20% of known pregnancies end in miscarriage,^[^ ^120^ ^]^ and 73 million elective abortions take place worldwide each year.^[^ ^121^ ^]^ Recent studies indicate that fetal cell transfer to the mother is more frequent and of higher quantity in pregnancies affected by placental dysfunction, which is a common feature of many adverse pregnancy outcomes.^[^ ^14^, ^15^, ^16^, ^18^ ^]^

While most microchimeric cells are acquired during pregnancy, breast milk provides another pathway for maternal cells to transfer to the neonate postpartum. Through diapedesis, these maternal cells migrate through the basement membrane via the paracellular pathway to enter the milk.^[^ ^122^, ^123^ ^]^ The neonatal intestinal mucosa is more alkaline than that of adults^[^ ^124^ ^]^ and highly permeable shortly after birth, making cell transfer from breast milk into offspring tissues potentially more likely before gut closure occurs.^[^ ^125^ ^]^ Studies across multiple animal species have demonstrated the transfer of maternal cells from breast milk to pups via the gut mucosa (reviewed in^[^ ^125^ ^]^). In human breast milk, various maternal cell types, including hematopoietic stem cells, mature immune cells, and mammary epithelial cells, have been identified, with their highest concentrations found in colostrum compared to mature milk.^[^ ^126^ ^]^ More recently, Balle and colleagues^[^ ^29^ ^]^ found that exclusive breastfeeding was associated with elevated levels of maternal microchimerism in the infant. These maternal cells, particularly breast milk stem cells, may contribute to the establishment of long‐term maternal microchimerism in infants during the early phase of lactation.

Related questions posed by the experts

**Tissue‐dependent microchimerism trafficking**

- What is the host origin (e.g., bone marrow, peripheral blood, breastmilk) and tissue distribution of microchimeric cells?
- In what form and in which niche can fetal microchimeric stem cells be found?
- Pregnancy‐acquired microchimerism can be extended by breast feeding, and milk‐associated maternal cells may also enter the neonatal circulation. Does breast‐feeding‐induced microchimerism affect gut colonization of offspring? How does third‐party milk, e.g. provided by milk banks, affect the acquisition of microchimeric cells (non‐identical/non‐related cells)?
- Does the function of maternal microchimerism vary according to mechanism of transfer (transplacental versus breastfeeding) or by tissue distribution?

**Cell transmission**

- What are the mechanisms for recruitment of fetal cells from maternal stem cell niches, once acquired during pregnancy?
- Is the transfer from host to host active or passive?
- Does the lineage/origin of the microchimeric cells matter for organ specificity?

#### 3.1.3 Subtheme: Persistence of Microchimerism

Persistence refers to the length of time microchimeric cells are sustained in the hosts and their long‐term effects (Table 2).

One key question is how microchimeric cells are able to persist in the body for long periods of time. Various factors may contribute to their persistence, including immune tolerance, cellular niches, and the ability of these cells to proliferate and integrate into host tissues. As previously mentioned, microchimeric cells of both fetal^[^ ^7^ ^]^ and maternal origin^[^ ^8^, ^42^ ^]^ have been detected in their respective hosts decades after pregnancy/birth, thus demonstrating the ability of these microchimeric cells to persist. Furthermore, debates concerning whether the requirement for long‐term persistence should be included in the definition of microchimerism (see Section 3.6) are ongoing.

The retention of fetal cells in maternal tissues seems to be conserved across mammalian species,^[^ ^127^, ^128^, ^129^, ^130^ ^]^ suggesting there are biological benefits to maintaining fetal tolerance after birth.^[^ ^129^, ^131^, ^132^ ^]^ As fetal microchimeric cell numbers acquired by the mother increase during gestation, immune tolerance to fetal antigens also increases with the expansion of maternal immunosuppressive T regulatory cells.^[^ ^133^ ^]^ This may reinforce tolerance of paternal antigens for future pregnancies, for instance, where the incidence of preeclampsia is reduced during second pregnancies with the same partner.^[^ ^134^, ^135^ ^]^ Cellular niches may also contribute to the persistence of fetal microchimeric cells: in one study, fetal mesenchymal stem cells were engrafted in maternal bone marrow and detectable decades later by immunocytochemistry and XY‐FISH in bone biopsies.^[^ ^136^ ^]^

In further support of immune tolerance of maternal microchimeric cells, exposure to non‐inherited maternal antigen (NIMA) in utero primes fetal T regulatory cells that mediate tolerance to maternal alloantigens^[^ ^46^, ^67^ ^]^ The persistence of maternal cells in fetal lymph nodes corresponds with ongoing maternal antigen tolerance that lasts at least through early adulthood.^[^ ^67^ ^]^ Similar links between expanded T regulatory cells with NIMA‐specificity have been described in mice.^[^ ^46^, ^137^ ^]^ Interestingly however, expanded accumulation of regulatory T cells can happen independently of overall maternal microchimerism levels. Depleting maternal microchimerism specifically from LysM+ CD11c+ cells eliminates NIMA‐specific FOXP3+ cell expansion without significantly reducing total maternal microchimerism.^[^ ^138^ ^]^ It is possible that postnatally, NIMA‐specific tolerance contributes to Rhesus factor (Rh) antigen sensitization in Rh‐negative women born to Rh‐positive mothers,^[^ ^139^ ^]^ however exposure to soluble Rh during fetal development may also contribute to this Rh‐specific tolerance. NIMA‐specific tolerance also limits antibody formation against NIMA in transfusion‐dependent individuals, even when they develop antibodies to most other HLA alloantigens.^[^ ^140^ ^]^ Therefore, postnatal persistence of NIMA‐specific tolerance may play a key role in how these cells persist long term. Likewise, the persistence of these cells is necessary for NIMA‐specific tolerance to persist, as has been shown for NIMA‐specific regulatory T cells lost following depletion of maternal microchimeric cells in mice.^[^ ^141^ ^]^ It was also found that retention of maternal microchimeric cells in female offspring promoted accumulation of fetal regulatory T cells with NIMA specificity,^[^ ^141^ ^]^ likely developed while exposed to maternal tissues in utero and early postnatal maturation. In the female offspring, these NIMA‐specific tolerant T regulatory cells were able to proliferate during pregnancies sired by males expressing alloantigens with overlapping NIMA specificity. These studies demonstrate that the acquisition and maintenance of NIMA tolerance may have impact on cross‐generational fitness (e.g., the success of second generation pregnancies).

Related questions posed by the experts

- What are the mechanisms that allow these foreign cells to persist?
- Is tolerance against microchimerism actively induced?
- Why are “foreign” microchimeric cells not eradicated by the immune system, and which escape mechanisms do they use?

#### 3.1.4 Subtheme: Quantity of Microchimerism

**Quantity of microchimerism** refers to the amount of microchimeric cells that may influence health during, but not limited to, pregnancy.

Microchimeric cells are detectable, but it is unclear if there is a threshold quantity of microchimeric cells that are needed to influence certain outcomes. It is known that fetal microchimerism increases in the maternal bloodstream during pregnancy, peaks at birth, and declines postpartum, with small numbers integrating into maternal tissues, and sometimes persisting for decades.^[^ ^30^ ^]^ However, less is known about the dynamic nature of microchimerism. For instance, is fetal microchimerism is sustained at elevated levels post‐partum, or for years post‐partum?

Parity does not seem to play a role in fetal microchimeric cell prevalence and concentration.^[^ ^142^ ^]^ In murine pregnancies, significant quantities of fetal microchimeric cells have been detected in the maternal lungs, spleen, liver, and kidneys.^[^ ^143^ ^]^ A similar distribution was also observed in the organs of women who died while pregnant or shortly after delivery of a son, where the lungs contained the highest amount of fetal microchimeric cells, followed by the spleen, liver, kidneys, brain, and heart through the detection of Y‐chromosome specific cells.^[^ ^144^ ^]^ These studies suggest that lungs may harbor higher quantities of fetal microchimeric cells due to their high blood flow and their hosting the first capillary bed through which blood from the placenta passes after entering uterine venous circulation. Interestingly, fetal microchimeric cells have been shown to include both parenchymal and hematopoietic cells.^[^ ^144^ ^]^ However, the quantity of cells may not necessarily influence health outcomes. Studies on a Danish cohort that tested for the presence or absence of male microchimerism, regardless of level, showed that women testing positive for microchimerism had higher survival than microchimerism‐negative women when deaths were due to cancer and cardiovascular disease.^[^ ^145^ ^]^

Additionally, microchimeric cells have shown preferential homing to sites of injury, which could influence the quantity and phenotype depending on disease status of the host.^[^ ^146^, ^147^, ^148^, ^149^ ^]^ Even at physiologically low levels, microchimeric cells can profoundly affect health. For instance, microchimeric cells can bring the HLA genetic susceptibility to developing RA to women with this disease who do not carry susceptibility genes in their own genome.^[^ ^90^ ^]^ In addition, as shown in a mouse model, low levels of microchimeric cells have the capacity to produce detectable RA‐specific autoantibodies.^[^ ^92^ ^]^ Interestingly, certain infections can also influence microchimerism quantity. For example, both peripheral^[^ ^58^ ^]^ and placental malaria^[^ ^28^ ^]^ were found to increase levels of maternal microchimerism in infants born to infected women. Additionally, Balle et al.^[^ ^29^ ^]^ found that maternal microchimerism was positively associated with the absence of maternal HIV, maternal and infant HLA class II compatibility, infant female sex, and exclusive breastfeeding in a cohort of infants from South Africa, and that maternal microchimerism was associated with infant T cell responses to vaccination. Following this work, Armistead et al.^[^ ^57^ ^]^ found that infants exposed to HIV, but uninfected, had lower levels of maternal microchimerism, specifically with a reduced level of maternal microchimeric CD8 T cells.

Related questions posed by the experts

- How does HLA compatibility play a role in quantity and distribution of microchimeric cells in host tissues?
- What impacts the amount (quantity) of microchimerism transferred transplacentally to the mother during pregnancy (fetal microchimerism) and to the offspring (maternal microchimerism)?
- Is there a minimum required microchimerism transfer level needed during pregnancy for optimal outcomes?
- How do small numbers of microchimeric cells impact host biology, and does the impact vary by microchimeric cell type?
- How does the difference (of whatever kind) of the parental immune systems impact the presence and biology of microchimeric cells?

#### 3.1.5 Subthemes: Cell Interaction and Plasticity

**Cell interaction and plasticity** are themes that have low representation, suggesting that these important perspectives on microchimeric cell function may be overlooked despite their potential significance. (Table 2). These subsections were combined and include how cells interact with their neighboring cells, adapt to external pressures, and have functional consequences for both mother and offspring.

Microchimeric cells can also demonstrate remarkable plasticity, differentiating into various tissue types as observed in mouse studies. Shao et al.^[^ ^150^ ^]^ found that maternal microchimeric cells are displaced by fetal microchimeric cells after the first pregnancy, and preexisting fetal microchimeric cells retained in dams are susceptible to displacement and replacement by new fetal microchimeric cells after subsequent pregnancies. The interactions of each generation with the host, and with pre‐existing fetal microchimeric cells may be beneficial or detrimental to an individual. This was recently demonstrated by Pham et al.,^[^ ^151^ ^]^ in which maternal microchimeric cells capable of producing C3 protein can partially override *E. coli* infection susceptibility in complement deficient offspring. This is in line with previous mouse studies showing that maternal microchimerism replaced IL‐2 in IL‐2 deficient offspring,^[^ ^152^ ^]^ and was a source of IgG‐secreting cells in offspring genetically deficient in B cells.^[^ ^153^ ^]^ These findings have implications for humans as they may explain why individuals with certain genetic disorders may present a spectrum of disease severity, due to postnatally retained functional maternal microchimeric cells.

Related questions posed by the experts

**Cell interaction**

- How do microchimeric cells interact with other cells, the host immune system, and with other sources of microchimerism within the same individual?

**Plasticity**

- How does a woman's microchimerism status fluctuate over her lifetime?
- In what ways do characteristics of microchimeric cells change in an individual over time?

### 3.2 Theme: Microchimerism in Interventions, Treatment and Transplantation

The theme, “Microchimerism in interventions, treatment and transplantation” describes questions revolving around the potential therapeutic applications of microchimerism (Table 2). While these questions imply functionality, these questions were categorized based on their relevance to clinical applications. The type of microchimerism discussed is not limited to pregnancy‐acquired microchimerism.

Several studies have examined the association of both fetal and maternal microchimerism in beneficial roles, as well as diseased states, such as autoimmune diseases^[^ ^66^, ^154^ ^]^ and cancer.^[^ ^23^ ^]^ Since pregnancy‐acquired microchimeric cells can persist for many years postpartum, it has been suggested that these cells may retain stem cell‐like properties.^[^ ^7^, ^8^ ^]^ For instance, stem‐like microchimeric cells^[^ ^131^ ^]^ may assist maternal tissue repair, where they have differentiated into organ‐specific phenotypes seen in some patients with thyroid or liver damage.^[^ ^75^, ^155^, ^156^ ^]^ Further observations have also shown stem‐like microchimeric cells developing endothelial phenotypes, contributing to vascularization in the tissues in which they engraft.^[^ ^157^, ^158^ ^]^ These findings suggest the possibility of developing therapeutic strategies that exploit the natural multilineage potential of microchimeric cells to promote tissue repair as well as to predict and identify risks for pregnancy complications,^[^ ^15^, ^85^, ^159^ ^]^ and autoimmune disease later in life.^[^ ^66^ ^]^

As microchimeric cells are tolerated in the host, there is a potential to utilize this mechanism in the field of transplantation to increase survival rates. Studies have observed that transplantation associated microchimerism in humans can be associated with graft acceptance,^[^ ^160^ ^]^ and lower rates of rejection after kidney, liver or small bowel transplantation.^[^ ^161^, ^162^ ^]^ In kidney transplantation, graft survival was significantly higher in recipients of kidneys from siblings expressing NIMA compared to siblings expressing inherited paternal antigens.^[^ ^163^ ^]^ This suggests that microchimerism may contribute to immune tolerance, or at least be indicative of it. However, it is important to also consider the quality and quantity of NIMA exposure as this may also contribute to an immunogenic response.^[^ ^164^ ^]^ There is also indirect evidence that maternal microchimeric cells found in cord blood grafts, which are sensitized to fetal inherited paternal antigens during pregnancy, may reduce the risk of leukemia relapse.^[^ ^165^ ^]^ Accounting for microchimeric NIMA exposure in patients and reproductive history in women (exposure to inherited paternal antigens) prior to transplantation would be relevant criteria to include when matching unrelated donors to recipients.^[^ ^166^, ^167^ ^]^ More work is needed to examine inter‐patient variability and differences in the subtype of microchimeric cells that are exchanged^[^ ^168^ ^]^ in order to advance medical interventions and the field of transplantation.

Related questions posed by the experts

- How can the therapeutic potential of microchimerism be harnessed (e.g., cell therapy for transplantation or fertility)? What are the long‐term health implications of microchimerism from medical interventions?
- Can we use processes related to fetal‐maternal microchimerism to optimize transplant survival rates?
- Is blood‐based detection an adequate marker of microchimerism status in women?
- Can it be confirmed that pregnancy‐acquired microchimerism is akin to a dormant stem cell engraftment?
- When in the life of the mother or offspring do these dormant cells, such as microchimerism acquired from blood transfusions, become functionally relevant?
- Does maternal microchimerism contribute to hematopoiesis in the bone marrow? Can microchimerism shape our lymphoid and myeloid landscapes?

### 3.3 Theme: Evolution

The theme “Evolution” relates to how and why microchimeric cells exist and persist using an evolutionary lens. While it is related to the functional properties of microchimerism, this theme is distinct as it considers potential adaptive properties. Its subthemes are broken down into adaptation and comparative approach (Table 2).

A key interest is in understanding how microchimeric cells contribute to human health and disease particularly during pregnancy and as a legacy of pregnancy. Microchimerism is known to play a paradoxical role in host health: sometimes beneficially, at other times detrimentally, e.g. in autoimmune disease^[^ ^66^, ^154^, ^169^ ^]^ and cancer.^[^ ^23^ ^]^ Turning to the principles of evolution, we can generate a cohesive framework to guide testable predictions, rationale, and methods in microchimerism research and their applications to human health and disease. An evolutionary perspective explores why humans are vulnerable to disease and accounts for environmental changes, lifestyle factors, and traits shaped by evolution.^[^ ^170^, ^171^ ^]^ Key questions posed by microchimerism experts can be described using Tinbergen's four questions to offer complementary perspectives to animal behavior.^[^ ^172^ ^]^ Bateson and Laland^[^ ^173^ ^]^ have since updated Tinbergen's questions to encompass both living and non‐living systems to help determine fitness relevant behaviors and traits for a given time and place. Thus, these questions can be broadly expressed within microchimerism research as: *why* do these cells exist and how do they contribute to overall fitness (function or adaptation); *when* did microchimerism evolve during our evolutionary history (evolution or phylogeny); *how* do they traffic between different organ and tissue types (causation or mechanism); and *how* do these cells shape the growth and development of the fetus (development or ontogeny). The framing of these questions depends on the source of microchimerism (e.g., maternal, fetal, donor‐specific) and context (e.g., pregnancy, transplantation, competitive evolutionary advantage).

Boddy and colleagues^[^ ^5^ ^]^ used these evolutionary principles to propose a cooperation and conflict framework for studying microchimerism. Reproductive success and survival is necessary for evolution to occur, but can come at a cost, a so‐called evolutionary trade‐off, when genetic interests diverge. Maternal‐fetal conflict theory^[^ ^174^ ^]^ is an extension of evolutionary theory and predicts that the consequences of escalated conflict between maternal and fetal interests could surpass a threshold leading to non‐optimal outcomes for both parties, resulting in pregnancy complications and disease in either individual.^[^ ^5^, ^175^, ^176^ ^]^ An evolutionary approach stemming from maternal‐fetal conflict theory can explain when and how microchimerism benefits hosts and when it may be harmful. However, we caution against viewing the role of microchimerism as a helpful/harmful dichotomy, but rather as a complex interaction between two genetically distinct individuals in which context matters.

Related questions posed by the experts

**Adaptation**

- Can genetic conflict between microchimeric cells and host cells be reduced in order to improve maternal and fetal outcomes?
- Do fetal pregnancy‐associated progenitor cells have a competitive advantage over innate maternal stem cells?
- What is the adaptive or evolutionary value of microchimerism?
- Which “evolutionarily paradoxical phenotypes” are caused by microchimerism?

**Comparative approach**

- Which animals experience bidirectional microchimerism? Does the type of placenta influence the transfer of cells?
- How is microchimerism distributed in the animal kingdom in general beyond humans and placental mammals?

### 3.4 Theme: Mapping “Generational Microchimerism”

The theme Mapping “Generational Microchimerism” describes functional and mechanistic questions between and across generations. This includes dyadic relationships between mother and fetus. The sub‐themes, intergenerational effect and transgenerational effect were used to distinguish microchimeric effects within and across generational lineages, respectively (Table 2).

The idea that mammals and other species can harbor microchimeric cells over generations (even transgenerationally) has been coined the “microchiome”.^[^ ^31^ ^]^ Yet, many studies suggest that humans are all transgenerational chimeras ‐ hosting cells from our grandmothers, mothers, older siblings, twins, and maternal aunts and uncles. This is based on direct evidence of microchimerism from close kin (e.g.,^[^ ^8^, ^44^, ^46^, ^82^, ^177^, ^178^ ^]^) and extrapolated from a subset of pivotal studies proposing broader patterns of microchimerism transfer.^[^ ^178^, ^179^ ^]^ Our understanding of generational microchimerism is limited by current analytical methods, specifically, the ability to identify trace numbers of microchimeric cells in hosts. Only recently has it been possible to understand which cells are sustained during pregnancy. Evidence from mouse models found that fetal microchimeric cells from previous pregnancies persist in subsequent pregnancies, allowing for maternal immune tolerance.^[^ ^150^ ^]^ This same study also found that maternal microchimeric cells present in daughters are replaced by fetal microchimeric cells once daughters become pregnant, complicating preexisting notions around generational microchimerism.^[^ ^150^ ^]^ The notion that older sibling or maternal cells can persist in pregnant females and have the potential to migrate to the developing fetus can inform genetic conflict studies and provide insight into disease vulnerability.

Further, evidence of other potential mechanisms of generational microchimerism are limited. How microchimeric cells are acquired, transferred, and sustained particularly within the context of breastfeeding is uncertain. While animal models have shown that maternal cells are present in the breast milk composition and can traffic to fetal tissue, evidence in humans is challenging to accumulate due to biological and environmental determinants (reviewed in^[^ ^125^ ^]^). For instance, variables like pathogen exposure that can alter breastmilk composition make it difficult to establish consistent findings across studies (see “cell transmission and tissue‐dependent trafficking” section below for a more detailed explanation).

Related questions posed by experts

**Intergenerational effect:**

- In multiparous mothers, do some pregnancies contribute more microchimerism than others, if so, why?
- Do human infants also acquire maternal microchimerism via breastfeeding? What is the impact of maternal microchimerism in human breast milk?

**Transgenerational effect:**

- Do we see multi‐generational transfer of microchimerism ‐ and what is the impact of these different cell lineages?
- What is the frequency of second‐order (e.g., siblings) and higher‐order (e.g., grandparent) microchimerism?
- Is generational microchimerism another mechanism to explain whether grandparent microchimerism impacts fetal development? If so, how?
- How can we identify the cellular composition and niches of residence within the microchimeric cell populations?

### 3.5 Theme: Microchimerism Detection

The theme “Microchimerism Detection” addresses the methodological challenges and technological advances needed to reliably identify microchimeric cells (Table 2). This includes multiple complementary techniques ‐ each with distinct advantages and limitations ‐ that capture their presence, spatial context, and biological function.

Microchimerism research has been limited by the lack of reliable detection methods, primarily due to the absence of suitable markers that could effectively be used to target these rare cells. Much of the existing knowledge comes from studies focused on male offspring ‐ detecting maternal (XX) cells in male (XY) samples or conversely, detecting male (XY) cells in female (XX) samples ‐ creating a sex‐biased understanding of microchimerism.^[^ ^180^, ^181^ ^]^ Below we discuss limitations and questions around methods to detect microchimerism.

PCR has historically been one of the primary methods used in microchimerism research, however, applications of this approach include detection biases based on sex and ancestry, and HLA compatibility. One of PCR's main limitations is the use of sex‐specific markers, specifically the *SRY* gene on the Y‐chromosome to detect microchimerism. The data PCR produces is also qualitative, indicating only the presence or absence of microchimerism, and lacks spatial resolution.^[^ ^180^, ^182^ ^]^ In recent years, quantitative PCR (qPCR) and droplet digital PCR (ddPCR) has circumvented some of these limitations by measuring microchimerism beyond Y‐chromosome markers. This was made possible by Lo and colleagues,^[^ ^183^ ^]^ who used qPCR to quantify cell‐free fetal DNA in maternal blood for non‐invasive prenatal testing. This technology was further adapted by targeting mismatches in the HLA loci between individuals to study microchimerism in autoimmune diseases such as scleroderma,^[^ ^101^ ^]^ rheumatoid arthritis^[^ ^90^ ^]^ and leukemia.^[^ ^184^ ^]^ While promising, the use of qPCR is limited in a few important ways. Cases in which no informative HLA mismatches exist require non‐HLA‐based assays^[^ ^185^ ^]^ such as targeting SNPs and insertion‐deletions, with assays often designed based on European population genetics. This highlights the need for assay expansion to ensure applicability across non‐European populations. Moreover, the validation and standardization of qPCR assays have been challenging due to limited availability of suitable positive control samples. Lastly, while qPCR quantifies DNA rather than viable cells, it does not capture the functional activity or phenotype of intact microchimeric cells in vivo, complicating interpretations of their biological impact (*49*).^[^ ^66^ ^]^

Other methods have attempted to examine the spatial context in which the microchimeric cells are located, using Fluorescence In Situ Hybridization (FISH) and immunofluorescence,^[^ ^21^, ^87^, ^181^ ^]^ however, these are often less sensitive. FISH enables tissue‐specific localization of microchimeric DNA by visualizing specific DNA sequences, targeting repetitive regions on the X and Y chromosomes. However, FISH is generally less sensitive than PCR and, similarly to PCR, is sex‐biased. In addition, XY‐FISH has been shown to yield false positive results, for example due to overlapping nuclei or signal artifacts, but this can be improved by reverse XY‐FISH, which inverts the typical detection setup to reduce background signals and improve specificity.^[^ ^186^, ^187^ ^]^ Another challenge of immunofluorescence is that it does not directly identify microchimeric cells but complements this by targeting protein markers to study cellular functions and interactions within the tissue microenvironment.^[^ ^78^, ^188^ ^]^ Immunohistological methods are limited by the availability of suitable antibodies, potential cross‐reactivity, and restrictions imposed by standard microscopy platforms, which confine the number of antibodies that can be used to the available fluorophores. They cannot be used for rare cell analysis without additional complementary validation methods. However, it might be useful to positively or negatively enrich a microchimeric target cell population for single‐cell laser capture microdissection followed by downstream analysis such as short tandem repeat (STR) typing, allowing to identify the cells’ origin.^[^ ^189^, ^190^ ^]^

Other methods based on antibody detection of non‐shared antigens (typically HLA class I molecules), such as Fluorescence‐Activated Cell Sorting (FACS), Magnetic‐Activated Cell Sorting (MACS), and Cytometry by Time‐Of‐Flight (CyTOF), share the limitations mentioned above. CyTOF, which uses heavy metal‐labeled antibodies instead of fluorophores, offers the advantage of simultaneously detecting a larger number of targets compared to FACS, enabling detailed phenotypic characterization.^[^ ^191^ ^]^ However, CyTOF ionizes the sample during processing, making it incompatible with further analysis. In contrast, FACS and MACS allow for the pre‐enrichment of microchimeric cells, which involves isolating cell populations of interest for further analysis. This pre‐enrichment enables downstream functional analyses such as culturing or sequencing. Nonetheless, these methods can provide valuable insights into the functional and biological roles of microchimeric cells.^[^ ^27^, ^192^ ^]^

Approaches that can identify unique microchimeric cells individually, like single‐cell RNA sequencing (scRNA‐seq) are gaining traction in microchimerism research. The advantage of scRNA‐seq is that it undergoes an unbiased gene expression profiling at the single‐cell level to produce a transcriptomic snapshot of individual cells. This detailed molecular information can precisely characterize microchimeric cells, providing information on their functional and biological role.^[^ ^71^, ^193^ ^]^ However, scRNA‐seq is resource‐intensive, requiring specialized tissue collection, storage, and preparation protocols, along with advanced computational resources and bioinformatics expertise to process large datasets and identify rare microchimeric cells within predominantly host cell populations.

Novel spatial analysis techniques, such as spatial transcriptomics, proteomics and metabolomics have been developed that provide multi‐omics data and spatial information.^[^ ^194^, ^195^, ^196^ ^]^ This will allow us to visualize where microchimeric cells are localizing within tissues and provide a detailed characterization of these cells. In addition, multi‐omics approaches increase the accuracy of the detection and provide more information about the function of the cells. These approaches are, similarly to scRNA‐seq, presently resource intensive, expensive, and require specialized sample preparation and analysis. Further, current approaches mostly use a targeted microarray approach, assaying approximately 500 genes, and thus may not be able to identify specific microchimeric cells. Likely in the future, it will be easier to use unbiased sequencing approaches. However, like single cell sequencing, the information from each individual cell may still be limited.

In addition to challenges of available technology, protocols for each of these techniques with respect to microchimerism analysis have not yet been standardized across laboratories. This lack of standardization may result in inconsistent sample collection and processing, variability in detection methods, and differences in data analysis that could introduce biases. Furthermore, no guidelines specifying which detection method is best suited for particular research questions currently exist.

Related questions posed by the experts

- How can we develop reliable and sensitive technologies to analyze and characterize microchimerism?
- How can we determine the individual source of microchimeric cells?
- What are the best methods for the unequivocal identification of microchimeric cells?
- What is the most efficient and reliable approach to detect and confirm fetal microchimerism, including distinguishing between multiple pregnancies?
- How can we best detect cellular ancestry, and how old is our oldest cell?

### 3.6 Theme: Definition of Microchimerism

The theme “Definition of Microchimerism” was not heavily endorsed at the time of the study (Table 2). Yet, open debates are currently unfolding as new knowledge emerges. Given its growing relevance, a clear definition is crucial and deserves to be established as its own theme. Traditionally, microchimerism has been understood as the harboring of a small amount of non‐self‐genetic material, typically acquired during pregnancy through a bidirectional exchange. This transfer of non‐self‐genetic material (i.e., cells or cell‐free DNA) occurs during each pregnancy between mothers and offspring, allowing for various multigenerational sources of microchimerism to co‐exist within an individual.^[^ ^197^ ^]^

However, in addition to pregnancy‐acquired microchimerism, there are other routes of acquiring microchimeric cells, such as iatrogenic microchimerism following organ transplantation or blood transfusion,^[^ ^160^, ^198^ ^]^ naturally through simultaneous or sequential siblings during pregnancy,^[^ ^82^ ^]^ or via lactation.^[^ ^125^ ^]^ As many physiological scenarios result in microchimerism, some may argue that only the temporary presence of genetically different cells or material is required, whereas others insist on the continued persistence or engraftment of microchimeric cells in the host.^[^ ^7^, ^8^ ^]^ Further discoveries such as cell‐free fetal DNA in the maternal circulation^[^ ^183^ ^]^ and extracellular vesicles of non‐host origin^[^ ^199^, ^200^ ^]^ also pose challenges to what is considered microchimerism. Complicating this issue even further are the unknowns of the functional and metabolic activity of microchimeric cells, with some debating that only metabolically active and proliferative cells should be classified as microchimerism. As it stands, this relatively young field requires a definition that remains fluid as more evidence emerges.

A note of clarification is needed here about the terminology and use of the word “fetal microchimerism.” Several studies have historically used samples from male offspring due to limited detection techniques (i.e., Y‐chromosomes in mothers indicate male‐origin cells). However, the presence of Y‐chromosome‐positive cells in women doesn't necessarily confirm fetal origin, since male microchimerism can arise from multiple sources. Male‐origin microchimerism has been found in women and girls that have never been pregnant,^[^ ^178^ ^]^ suggesting not all male microchimerism is of fetal origin. We also cannot rule out other possibilities such as microchimerism of sibling origin (i.e., cells from older brothers, or twins) and males from maternal lineage (i.e., cells from maternal uncles or grandmothers). Despite its high multilineage potential, “fetal microchimerism” typically refers to fetal origin cells that are acquired during pregnancy.

Related questions posed by the experts

- Is microchimerism defined by the ability to persist long‐term and the stem cell nature of the acquired cells?
- Does microchimerism also encompass more temporary cells that persist for a while in the maternal body, but that do not engraft, such as fetal erythrocytes or placental trophoblasts that are transferred to the mother during pregnancy?

### 3.7 Theme: Appropriate Experimental Model Systems

The last theme, “Appropriate Experimental Model Systems,” had the smallest percentage of questions asked (Table 2). This question did not easily fall under the “Microchimerism Detection” theme. While related, it touches on separate issues. This theme delves specifically on study design and choosing appropriate animal models and less on the technology to detect microchimerism.

Murine models are widely used in microchimerism research due to their genetic malleability, relatively short reproductive cycles, and the extensive range of tools available for genetic manipulation. These factors make mice ideal for studying the persistence of fetal cells postpartum,^[^ ^201^ ^]^ their phenotypic diversity across maternal tissues,^[^ ^202^ ^]^ and their functional roles in maternal tissue during and after pregnancy.^[^ ^127^ ^]^ Tracing maternal cell migration and exploring its impact on the developing immune system are some of the main advantages of murine models.^[^ ^27^, ^47^, ^71^, ^127^, ^203^, ^204^ ^]^ However, significant differences in placentation, litter size, and genomic backgrounds between humans and mice limit the translational relevance of murine findings to human microchimeric processes.

Others have utilized nonhuman primates to model immune responses and disease progression.^[^ ^130^, ^205^ ^]^ While nonhuman primate models hold substantial potential due to their close genetic and physiological similarities to humans, practical limitations including ethical concerns, high costs, and complex logistics, continue to constrain their application in this research area. As with all experimental animal models, caution is needed when extrapolating findings to human contexts.^[^ ^144^ ^]^

Related questions posed by the experts

- Which animal models are most appropriate for studying the biological functions and disease roles of microchimerism, and for understanding human microchimerism?

## 4 Additional Research Challenges

Experts described major challenges related to the limitations in methodologies (**Table** **4**). For more details, refer back to Sections 3.5 and 3.7. See Table S4 (Supporting Information) for specific challenges. Additional challenges that received less attention but may be equally important to help advance the field, are described below. We note that some of the challenges described are general challenges of genomic data analyses and women's health research.

**Table 4:** Challenges with Microchimerism Research. Frequency of Main Themes and Subthemes. A total of 53 challenges were provided. Frequency of themes raised by authors is shown.

Challenges with microchimerism research themes	Subthemes	Frequency (of 100 %) of themes raised by authors
Complex diseases		2%
Ethical constraints		2%
No clear definition of microchimerism		2%
Diversity of microchimerism		4%
Lack of research community		4%
Limited interest in pregnancy, reproductive biology, women's health		4%
Young field		4%
Lack of grant funding		4%
Limitations in methodology		68%
	Lack of appropriate animal model	6%
	Uncontrolled experimental designs	6%
	Technology	20%
	Limited longitudinal data and challenging data collection	33%
	Microchimerism is rare and hard to detect	36%

### 4.1 Challenge: Lack of Grant Funding

Funding has been difficult to obtain in microchimerism research. The rarity of the cells can brew skepticism among grant reviewers and critics, especially because the functional impact of these cells on host biology is not well established. There has also been a historical underappreciation for funding women's health research as a result of historical gender bias in medicine and science, societal and cultural attitudes toward pregnancy and women, economic factors and profitability, research limitations, and accessibility, and government policy.^[^ ^206^, ^207^, ^208^ ^]^

### 4.2 Challenge: Lack of Research Community

The field of microchimerism research is young. There is a limited number of researchers who have the capacity to study these cells, as this research remains “high‐risk, high‐reward”. An integrated community of microchimerism researchers is needed to facilitate microchimerism research, to break through current technical and conceptual barriers, and to develop standardized practices to grow the research community. This review provides evidence for how the microchimerism community is joining forces to reach these goals.

### 4.3 Challenge: Ethical Constraints

The ethical constraints described are not unique to microchimerism research and can be applied to most of maternal‐offspring health research. Broad ethical concerns include, but are not limited to the study of pregnant populations and fetus; human biosample data collection; and data storage and sharing practices (i.e., genetic information).^[^ ^209^, ^210^ ^]^ More specific to microchimerism research are aspects that relate to feasibility and participant consent as microchimerism research often involves at least two participants. Standardized practices in microchimerism research grounded in the CARE (Collective Benefit, Authority to Control, Responsibility, Ethics) principles^[^ ^211^ ^]^ based on country‐specific standards are needed. Moving forward, the field must continue to balance these ethical considerations with the pursuit of knowledge that could benefit maternal and child health.

## 5 Concluding Remarks

The Microchimerism, Human Health and Evolution Project was formed to address two main goals. First, it was designed to understand the etiology of microchimerism and its role in health and disease. Second, it aims to address the methodological and theoretical constraints to studying microchimerism. Leading experts were called on to provide open questions in the field of microchimerism research. Notably, almost every surveyed person posed at least one question related to function and/or mechanism. More data generated regarding function and/or mechanism will help contribute to answering the questions laid out by experts and will further shape the field of microchimerism.

Our analysis revealed gaps within the current microchimerism research landscape. Questions related to evolutionary aspects, social determinants of health, and ethical considerations were notably underrepresented. While we engaged with an influential group of experts, we recognize that our study may be biased. The network of experts we consulted frequently collaborate with each other, which is common in specialized research areas but may limit exposure to diverse perspectives and methodological approaches. Notably, researchers from emerging fields or those publishing outside mainstream journals are likely underrepresented in our response group. Recognizing these limitations underscores the importance of broadening the research community to scholars from different disciplines and institutions. As such, the ultimate goal for creating this consortium is to build and grow the microchimerism research community by holding regular scientific meetings describing works in progress, creating more opportunities to train in research methods and techniques across different laboratories, create pipelines to allow for the sharing of protocols, and hold annual conferences to bring the community and those interested together. Overall, we believe that identifying and answering these questions will provide a foundation for advancing knowledge of microchimerism biology and health for the future.

## Conflict of Interest

The authors declare no conflict of interest.

## Author Contributions

K.J.C., R.C.Q., and K.S. contributed equally to this work. T.K. and A.M.B. are the co‐senior authors. A.M.B. and K.J.C. performed conceptualization. K.J.C., K.S., and R.C.Q. performed data curation. K.J.C., K.S., and R.C.Q. performed formal analysis. A.M.B., T.K., F.A.S., M.E., H.J.C., K.J.C., K.S., R.C.Q., I.A.S., and T.P. performed funding acquisition. C.A.A., P.A., D.W.B., H.D.C., H.J.C., M.E., H.E.S.F., D.H., W.E.H., W.H., D.P.J., M.K.‐J., S.B.K., K.K., N.L., J.L.N., M.B.O., T.P., J.R.P., F.A.S., A.C.S., A.S., I.A.S., C.U., S.S.W., M.A.W., J.Y., T.K., and A.M.B. performed investigation. K.J.C., R.C.Q., and K.S. performed methodology. K.J.C. performed project administration. A.M.B. and T.K. performed supervision. K.J.C. and R.C.Q. performed visualization. K.J.C., R.C.Q., and K.S. wrote the original draft. K.J.C., R.C.Q., K.S., C.A.A., P.A., D.W.B., H.D.C., H.J.C., M.E., H.E.S.F., D.H., W.E.H., W.H., D.P.J., M.K.‐J., S.B.K., K.K., N.L., J.L.N., M.B.O., T.P., J.R.P., F.A.S., A.C.S., A.S., I.A.S., C.U., S.S.W., M.A.W., J.Y., T.K., and A.M.B. performed wrote, reviewed, and edited the draft.

## Supporting information



Supplemental Table S1

Supplemental Table S2

Supplemental Table S3

Supplemental Table S4
