# Supplementary material for: Identifying Key Questions and Challenges in Microchimerism Biology
Source: Adv Sci (Weinh). 2025 Oct 24;12(48):e14969. doi: 10.1002/advs.202514969 (PMC12752559; doi:10.1002/advs.202514969)
Supplement: Supplementary file 1 — Supplemental Table S1 [file ADVS-12-e14969-s003.docx]

| Date | Pivotal discoveries and/or contributions to the field of microchimerism | Species | Type of chimerism or microchimerism | Sample used | Detection method(s) | Reference |
| --- | --- | --- | --- | --- | --- | --- |
| 1893 | German pathologist, Georg Schmorl, first documented the presence of fetally derived syncytial knots in lung capillaries of eclamptic women who died during pregnancy. | Human | Fetal MC | Lung | Histology Microscopy | ^[3]^ |
| 1916 | Study found that dizygotic twin cows often share blood circulation and hormones *in utero* by vascular anastomoses, leading to suppressed development of the reproductive system in the female twin (i.e., "freemartin”). | Bovine | Twin-twin chimerism | Uterus Ovary Placenta Fetal tissue | Anatomical examination | ^[32]^ |
| 1945 | Study contributed to the concept of immunological tolerance after finding that fraternal twin cows often exchange blood precursor cells, allowing mixed blood to persist in the adult animal (i.e., two distinct blood groups, their own and that of their twin). | Bovine | Twin-twin chimerism | Whole blood | Blood grouping Serology | ^[33]^ |
| 1951 | Most dizygotic cattle twins can accept skin grafts from each other, which is specific tolerance. Whereas third party skin transplants were rejected. | Bovine | Twin-twin chimerism | Skin graft | Observation | ^[1]^ |
| 1953 | Billingham et al. report that mice and chickens injected as embryos with cells from other strains acquire a persistent tolerance to later skin grafts from the donor strains. | Murine, chicken | Transplant chimerism | Skin graft | Observation | ^[34]^ |
| 1953 | Case study found blood donor with blood type O and A; type A came from her twin brother who died 3 months after birth. | Human | Twin-twin chimerism "blood group chimera" | Whole blood | Blood grouping Serology | ^[35]^ |
| 1954 | Study found Rh-negative women acquired Rh tolerance due to *in utero* exposure to maternal Rh-positive cells. | Human | Maternal MC | Whole blood | Blood grouping Rh-testing | ^[139]^ |
| 1969 | First report of chimeric cells in long-surviving recipients of orthotopic hepatic allografts detected by female Kupffer cells; persistence of donor-specific immunoglobulins and red blood cell alloantibodies. | Human | Transplant chimerism | Lymphoid Liver tissue | Hemagglutination inhibition test of Martensson Barr body staining | ^[36]^ |
| 1972 | Study identified the presence of circulating male leukocytes in the PB of women. | Human | Fetal MC | Peripheral blood | Quinacrine Y-chr stain | ^[37]^ |
| 1977 | The term “microchimerism” used to describe long-term donor cell survival in a small proportion relative to the host cell numbers. | Human | Transplant microchimerism |  |  | ^[4]^ |
| 1979 | Study found male fetal cells bearing unique paternally inherited cell surface antigens from maternal blood were detected at 15 weeks of gestation. | Human | Fetal MC | PBMC | FACS Quinacrine Y-chr stain Microscopy | ^[38]^ |
| 1980 | Study found that maternal cells engraft and persist in the circulation of infants with severe combined immunodeficiency. | Human | Maternal MC | Whole blood Skin biopsy | HLA typing Karyotyping | ^[39]^ |
| 1984 | Study found potential measurable immunologic benefit to the recipient of a subsequent maternal-donor related renal transplant following exposure to maternal lymphocytes via breast feeding during infancy. | Human | Breast milk maternal MC | Patient data | Family history Post-transplant evaluation | ^[212]^ |
| 1989 | Authors raised the possibility of whether maternal cells may enter fetal circulation and contribute to GVHD in UCB transplantation. | Human | Maternal MC | NA | NA | ^[213]^ |
| 1989 | PCR amplification used to detect male fetal cells from maternal peripheral circulation. | Human | Fetal DNA | PB DNA | Nested PCR of Y-chr | ^[40]^ |
| 1990 | Study detected a single-copy of fetal DNA sequence from maternal blood. | Human | Fetal DNA | PB DNA | Nested PCR of Y-chr | ^[214]^ |
| 1994 | Study detected male fetal DNA in maternal circulation at 4 weeks gestation. | Human | Fetal DNA | PB DNA | Nested PCR of Y-chr | ^[41]^ |
| 1996 | Study found that fetal cells can persist in maternal circulation for decades postpartum; full-term pregnancy is not required. | Human | Fetal MC | Venous blood  Venous blood DNA | FACS PCR for Y-chr | ^[7]^ |
| 1996 | Study found bilateral trafficking of nucleated cells between the fetus and the mother. | Human | Fetal MC Maternal MC | Maternal blood DNA UCB DNA | Nested PCR of Y-chr & polymorphisms | ^[42]^ |
| 1997 | Study detected maternal cells in fetal blood (obtained by intracardiac puncture) during the 3^rd^ trimester of pregnancy. | Human | Maternal MC | Fetal blood DNA Maternal blood DNA | PCR Southern blotting | ^[215]^ |
| 1998 | Study identified mMC in 2nd trimester fetal products of conception. | Human | Maternal MC | Fetal blood DNA Maternal blood DNA Amniotic fluid DNA | Nested PCR for polymorphisms | ^[216]^ |
| 1998 | Compared to non- preeclamptic patients, study found that fetal erythroblast numbers are elevated in the PB of preeclamptic patients. | Human | Fetal MC | Venous blood | MACS XY-FISH | ^[217]^ |
| 1998 | Study found association between increased Fetal MC and scleroderma; implications of HLA compatibility in scleroderma and other autoimmune diseases. | Human | Fetal MC | Whole blood DNA | qPCR for Y-chr | ^[97]^ |
| 1999 | Study found that mMC cells can persist in immunocompetent offspring into adult life (up to 46 years old). | Human | Maternal MC | PBMC DNA | HLA genotyping, PCR for HLA sequences, XY-FISH | ^[8]^ |
| 1999 | Study observed the persistence of pig MC in human xenotransplant patients for up to 8.5 years post-transplant. | Porcine | Xenotransplantation MC | PBMC DNA Saliva DNA | PCR for porcine-specific sequences | ^[218]^ |
| 2000 | Study found mMC Ig-secreting B cells can transfer to the mouse fetus postnatally via milk. | Murine | Maternal MC | Spleen Bone marrow | PCR Flow cytometry Confocal laser scanning microscopy | ^[153]^ |
| 2000 | Compared with DNA from the cellular fraction of maternal blood, study found more fetal DNA is present in the plasma of pregnant women ; maternal DNA found in both the cellular and plasma fractions of cord blood after delivery. | Human | Fetal MC Maternal MC | PBMC DNA Plasma DNA | Real-time qPCR for Y-chr *SRY* & non-HLA polymorphisms | ^[108]^ |
| 2001 | Study detected Fetal MC in maternal circulation at 7 weeks gestation. | Human | Fetal MC | Whole blood DNA Plasma DNA | Real-time kinetic-PCR for Y-chr | ^[111]^ |
| 2001 | Study found peak Fetal MC cells in maternal circulation at time of elective termination of pregnancy. | Human | Fetal MC | PB DNA | qPCR for Y-chr | ^[119]^ |
| 2002 | Study first found Fetal MC in salivary glands and lungs of female patients with Sjögren's syndrome. | Human | Fetal MC | PBMC DNA Labial salivary gland Bronchoalveolar lavage fluid | Nested PCR XY-FISH | ^[219]^ |
| 2003 | Study found Fetal MC cells to have a progenitor phenotype. | Human | Fetal MC | Whole blood Whole blood DNA | MACS Y-chr FISH Nested PCR for *ZFY* target | ^[220]^ |
| 2003 | Study found that fetal loss can influence persistence of Fetal MC cells. | Human | Fetal MC | Case reports Cohort studies with MC status | Detailed pregnancy history MC data reported | ^[221]^ |
| 2003 | Study found maternal cells enter fetal circulation and migrate to fetal organs. | Human | Maternal MC | Fetal liver Spleen Thymus Thyroid Skin | XY-FISH | ^[222]^ |
| 2004 | Study found Fetal MC cells in maternal tissues have multilineage capacities. | Human | Fetal MC | Archived paraffin-embedded: thyroid Cervix Gallbladder  Intestine Liver Spleen Lymph node Skin  UCB | XY-FISH Immunostaining Histology | ^[43]^ |
| 2004 | Study found Fetal MC stem cells transfer into maternal blood and engraft in bone marrow, where they remain throughout life. | Human | Fetal MC | Bone marrow Rib | XY-FISH | ^[136]^ |
| 2005 | Study identified Fetal MC male cardiomyocytes of extracardiac origin in the hearts of women with male progeny. | Human | Fetal MC | Heart tissue | Real time qPCR for Y-chr *SRY* XY-FISH IHC HLA typing | ^[12]^ |
| 2005 | Study found fetal CD34+ cells in the maternal circulation of nonhuman primate species persist in maternal tissues post-delivery. | Rhesus Monkey | Fetal MC | PBMC DNA DNA of Paraffin-embedded: Pituitary Lymph node Thyroid Lung Heart Liver Spleen Pancreas Adrenals Kidney Large intestine Skin | MACS Real-time qPCR for Y-chr *SRY* & *TSPY* | ^[132]^ |
| 2005 | Study demonstrated that male cells are present in normal kidneys, livers, and hearts of women without male progeny. | Human | Fetal MC | Archived paraffin-embedded: Kidney Liver Heart Spleen | XY-FISH Nested PCR of Y-chr *SRY* | ^[45]^ |
| 2005 | Study found that MC with male DNA in circulation of women who never gave birth to a son, suggesting alternative sources of MC. | Human | Male-origin MC | PBMC DNA | Real time qPCR for Y-chr *DYS14* | ^[44]^ |
| 2005 | Study detected male-origin MC in liver of normal and diseased female livers; presence of male cells in female fetuses and children potentially acquired transplacentally from previous pregnancies with male offspring. | Human | Male-origin MC | Paraffin-embedded liver Frozen liver Liver DNA | Nested PCR Real-time PCR for Y-chr Y-chr genotyping XY-FISH | ^[223]^ |
| 2007 | English translation of Georg Schmorl's work from 1893 with a critical re-evaluation of its conclusions from a 21st century perspective. | Human | Fetal MC | NA | NA | ^[2]^ |
| 2007 | Compared to unaffected siblings and healthy individuals, study found higher levels of mMC in their circulation; mMC cells contribute to islet beta cells in mother progeny. | Human | Maternal MC | Whole blood DNA mouth swab DNA Male pancreatic tissue | qPCR for HLA alleles XY-FISH | ^[102]^ |
| 2007 | Study found maternally-derived cells persist in the spleen and thymus, and can produce IL-2 sufficiently to alter the phenotype of IL-2 KO mice after correcting for deficiencies in fetal immune development. | Murine | Maternal MC | transgenic mouse thymus single-cell suspension | ISH IHC qPCR flow cytometry | ^[152]^ |
| 2008 | Study found mMC cells in 2^nd^ trimester fetuses and persist across tissues in multiple phenotypes. | Human | Maternal MC | Fetal tissue & DNA | MACS HLA typing PCR | ^[224]^ |
| 2008 | Study found that Fetal MC cells are not necessarily associated with disease and can travel to distant organs, thyroid, lung, skin, and lymph nodes in the maternal host. | Human | Fetal MC | Archived paraffin-embedded: Thyroid Lung Skin lymph node | XY-FISH Nested PCR of Y-chr *SRY* | ^[45]^ |
| 2008 | Study detected maternal antigen-presenting cells in fetal lymph nodes suggesting *in utero* education of the immune system to tolerate NIMA expressing maternal cells. | Human | Maternal MC | Fetal mesenteric lymph node Spleen Thymus Infant thymus PBMC DNA UCB DNA | Real-time qPCR: HLA-DR & InDel MACS Flow cytometry IHC FACS | ^[67]^ |
| 2010 | Study found fetal cells have hematopoietic potential, where lymphoid progenitor activity of fetal origin was demonstrated in mothers that had an immune deficiency. | Murine | Fetal MC | transgenic mouse maternal blood spleen bone marrow | FACS | ^[225]^ |
| 2010 | Study improved verification of detected MC cells demonstrating that XY-FISH is prone to result in false positives and that reverse XY-FISH and STR analysis allows unambiguous confirmation of MC status. | Human | MC “spike-in” | PBMC Y-chr positive choriocarcinoma cell line JAR first trimester decidua chorionic villous tissue | MACS Immunofluorescence Laser microdissection and subsequent laser catapulting Multiplex PCR | ^[189]^ |
| 2010 | Study found that early after implantation, fetal cells with multilineage potential migrate first to maternal bone marrow, and upon injury, migrate to damaged organs. | Murine | Fetal MC | Liver Kidney Pancreas Heart Lung Bone marrow | Histology Real-time qPCR | ^[226]^ |
| 2010 | Study found maternal and fetal origin CD66B^+^ cells have an active microchimeric hematopoietic stem and progenitor cell niche, which may impact innate and adaptive immune responses. | Human | Fetal MC Maternal MC | PB PBDNA | FACS qPCR for HLA alleles & Y-chr *DYS14* | ^[227]^ |
| 2012 | Study identified male MC DNA in human female brains and specific brain regions. | Human | Fetal MC | Brain tissue DNA | Real-time qPCR for Y-chr *DYS14* | ^[10]^ |
| 2012 | Study found Fetal MC is poorly predicted by pregnancy. | Human | Male-origin MC | Buffy coat DNA | Real-time qPCR for Y-chr *DYS14* | ^[228]^ |
| 2012 | Study found that the presence of male origin MC is associated with reduced risk of developing breast cancer. | Human | Male-origin MC | Buffy coat DNA | Real-time qPCR for Y-chr *DYS14* | ^[73]^ |
| 2012 | Study found fetal cells that enter maternal circulation during pregnancy may persist in maternal tissue for decades and aid in healing injured tissues. | Murine | Fetal MC | transgenic mouse maternal heart eGFP-tagged fetal tissue | Immunofluorescence XY-FISH | ^[158]^ |
| 2013 | Study detected Fetal MC by Y-chromosome in dogs following pregnancy. | Canine | Fetal MC | Whole blood DNA PBMC DNA | Nested PCR for canine Y-chr | ^[129]^ |
| 2013 | Compared to women without preeclampsia, study found women with preeclampsia have higher concentrations of Fetal MC. | Human | Fetal MC | PBMC | qPCR for HLA & non-HLA alleles | ^[85]^ |
| 2014 | Study found male MC presence in PB of women is associated with substantially improved survival in women. | Human | Male-origin MC | Buffy coat | qPCR for Y-chr *DYS14* | ^[145]^ |
| 2014 | Study found Fetal MC supports maternal wound healing. | Human | Fetal MC | Skin biopsy | Fluorescent IHC XY-FISH nested PCR for Y-chr *SRY* | ^[13]^ |
| 2014 | Study utilized sensitive flow cytometric detection of mMC cells in fetal organs by targeting two distinct markers, CD45.1^+^ and the H-2 complex. | Murine | Maternal MC | transgenic mouse maternal uterine-draining lymph nodes bone marrow fetal thymus bone marrow DNA | Flow cytometry qPCR | ^[229]^ |
| 2015 | Study found that maternal cells that establish MC in female offspring during development promote systemic accumulation of immune suppressive regulatory T cells with NIMA specificity, having long term impact on reproductive success. | Murine | Maternal MC | Heart Liver Uterus Prostate DNA | Tetramer enrichment and flow cytometry qPCR | ^[141]^ |
| 2015 | Study found Y-chromosome is frequently present in young girls. | Human | Male-origin MC | Buffy coat DNA | Real-time qPCR for Y-chr *DYS14* | ^[178]^ |
| 2015 | Compared to non-pregnant women, study found that tissue MC occurs more frequently in the lungs, spleens, livers, kidneys, and hearts of pregnant women. | Human | Fetal MC | Formalin-fixed, paraffin-embedded: Lung Spleen Kidney Heart Brain | ISH for Y-chr IHC | ^[144]^ |
| 2017 | Study found mMC influences neonatal immunity via *in utero* transfer. | Human | Maternal MC | UCB DNA | qPCR for HLA & non-HLA alleles | ^[28]^ |
| 2018 | Study found mMC levels are increased in preterm vs. term infant cord blood. | Human Murine | Maternal MC | Whole blood DNA UCB DNA | Real-Time qPCR for HLA-DR & non-HLA alleles Flow cytometry FACS | ^[230]^ |
| 2019 | Study detected Fetal MC in maternal hair follicles decades postpartum. | Human | Fetal MC | Eyebrow hair follicles | XY-FISH | ^[231]^ |
| 2019 | Study found that mouse mothers transfer antigen-specific T cell mMC cells via breastfeeding to provide offspring with long-term protection from parasitic infection. | Murine | Breast milk maternal MC | Milk bolus | Flow cytometry | ^[232]^ |
| 2019 | Study found persistent transfusion-associated MC is likely restricted to transfusion following traumatic injury. | Human | Transfusion-associated MC | White blood cell DNA | Real-time qPCR for non-HLA alleles | ^[233]^ |
| 2019 | Study demonstrated functional mechanism of Fetal MC to trigger rheumatoid arthritis in postpartum women. | Human | Fetal MC | PBMC DNA | qPCR for HLA & non-HLA alleles | ^[184]^ |
| 2021 | Study found that presence of male origin MC is associated with reduced risk of developing ischemic heart disease. | Human | Male-origin MC | Buffy coat DNA | Real-time qPCR for Y-chr *DYS14* | ^[234]^ |
| 2021 | Study found that male origin MC is associated with reduced risk of developing ovarian cancer. | Human | Male-origin MC | Buffy coat DNA | Real-time qPCR for Y-chr *DYS14* | ^[83]^ |
| 2021 | Study found transgenerational transfer of cells beyond mother-baby dyad, including grandmaternal cells in cord blood have implications in immunology and evolution. | Human | Grand maternal MC | UCB DNA Maternal grandmother whole blood DNA Maternal whole blood DNA | qPCR for HLA alleles | ^[179]^ |
| 2021 | Study found that mMC cells support hematopoietic stem cell differentiation in fetal bone marrow, and neonatal immunity via in utero and breast milk-derived transfer. | Murine | Maternal MC | transgenic mouse fetal bone marrow liver spleen thymus | MACS flow cytometry FACS | ^[27]^ |
| 2022 | Study identified role of progesterone in prompting Tregs for immune tolerance; importance of NIMA specific regulatory T cells in contributing to tolerance of mMC into offspring. | Mouse | Maternal MC | transgenic mouse spleen lymph node uterus decidua placenta | qPCR, tetramer enrichment flow cytometry | ^[235]^ |
| 2022 | Study found maternal infection influences the levels of mMC in children, which may subsequently affect infant T cell responses. | Human | Maternal MC | Whole blood DNA | qPCR for HLA & non-HLA alleles | ^[29]^ |
| 2022 | Study found mMC influences brain development and postnatal behavior. | Murine | Maternal MC | Fetal brain (embryonic day 18.5) postnatal brain (day 8) Adult brain (day 60) | Flow cytometry ScRNA-Seq | ^[71]^ |
| 2023 | Study found that following SARS-CoV-2 messenger RNA vaccination, maternal breast milk contains SARS-CoV-2 spike-specific T cells; implications for neonatal immunity. | Human | Breast milk maternal MC | Breast milk Buffy coat DNA | FACS Flow cytometry HLA typing | ^[236]^ |
| 2023 | Study found presence of male origin MC is associated with reduced risk of developing brain cancer. | Human | Male-origin MC | Buffy coat DNA | Real-time qPCR for Y-chr *DYS14* gene | ^[237]^ |
| 2023 | Study found Fetal MC occurs in greater frequency in women affected by preeclampsia with altered immune subsets. | Human | Fetal MC | PBMC DNA | qPCR for HLA-alleles | ^[86]^ |
| 2023 | Study found pre-existing Fetal MC are displaced by new Fetal MC of subsequent pregnancies in mice. | Murine | Preexisting MC | transgenic mouse tissue | qPCR, tetramer enrichment Flow cytometry | ^[150]^ |
| 2023 | Study found pathogen specific mMC in offspring can be activated and assist with early life infections. | Murine | Maternal MC | transgenic mouse fetal bone marrow spleen liver maternal uterus liver spleen uterus‐draining lymph nodes | MACS flow cytometry | ^[47]^ |
| 2024 | Study found mMC cells to be functional and secrete complement in complement deficient offspring. | Murine | Maternal MC | transgenic mouse tissue  serum  DNA | qPCR | ^[151]^ |

**Table S1. Historical overview of pivotal discoveries in the fields of immunology and transplantation contributing to the development of the microchimerism field.** Table highlights the contribution to the field, the species that the study used, sample type used, detection methods utilized to identify microchimerism and the original reference. Where “male-origin microchimerism” is stated, this indicates that the study was able to detect male DNA, but was unable to confirm fetal microchimerism due to a previous pregnancy where the offspring was male. Abbreviations are as follows: AF, amniotic fluid, Chr, chromosome; DNA, deoxyribonucleic acid; *DYS14*, alias of *TSPY* gene; eGFP, enhanced green fluorescent protein; FACS, Fluorescence-activated cell sorting; FISH, Fluorescence in situ hybridization; HLA, human leukocyte antigen; IHC, immunohistochemistry; InDel, insertion-deletions; ISH, In Situ Hybridization; MACS, Magnetic-activated cell sorting; mMC, MC, microchimerism; NIMA, non-inherited maternal antigen; PB, peripheral blood; PBMC, peripheral blood mononuclear cells; PCR, polymerase chain reaction; qPCR, quantitative polymerase chain reaction; RNA, ribonucleic acid; Rh, rhesus factor; SARS-CoV-2, severe acute respiratory syndrome coronavirus 2; ScRNA-seq, Single-cell RNA sequencing; *SRY*, sex determining region of Y chromosome gene; STR, short tandem repeats; *TSPY*, testes specific protein Y gene UCB, umbilical cord blood; *ZFY*, Zinc finger Y-chromosome gene.
