# Supplementary material for: Identifying Key Questions and Challenges in Microchimerism Biology
Source: Adv Sci (Weinh). 2025 Oct 24;12(48):e14969. doi: 10.1002/advs.202514969 (PMC12752559; doi:10.1002/advs.202514969)
Supplement: Supplementary file 2 — Supplemental Table S2 [file ADVS-12-e14969-s001.docx]

**SURVEY**

**Identifying key questions in microchimerism (MC) biology and health**

What is your name?

- First Name __________________________________________________
- Last Name __________________________________________________

What is the best email address to reach you?

________________________________________________________________

What is your author affiliation, for publication purposes?

- Department __________________________________________________
- University or other affiliation __________________________________________________
- Address __________________________________________________

What are the **pivotal discoveries** since the inception of the field of MC research? (300 words max)

Please provide references listed in your response.

________________________________________________________________

________________________________________________________________

________________________________________________________________

________________________________________________________________

What do you think are the **open questions** (list between 3 - 5 questions) that future MC research should prioritize in the next 20 years?
Please rank your questions by order of importance.

- Question #1 __________________________________________________
- Question #2 __________________________________________________
- Question #3 __________________________________________________
- Question #4 __________________________________________________
- Question #5 __________________________________________________

Please provide a **brief explanation** detailing **why the questions are of key importance** and **what kind of impact it** has on the field. While we are interested in your rationale for each individual question, feel free to combine and describe them broadly, short-term vs. long-term questions, etc. (300 words max)

________________________________________________________________

________________________________________________________________

________________________________________________________________

________________________________________________________________

What are **the reasons these questions remained unanswered**? Feel free to provide an explanation for each individual question or a general overview describing challenges in addressing these questions. (300 words max)

________________________________________________________________

________________________________________________________________

________________________________________________________________

________________________________________________________________

Please provide any additional **references** that you feel are important to include in this manuscript.

________________________________________________________________

________________________________________________________________

________________________________________________________________

________________________________________________________________

If you have any suggestions for potential target journals, please include them here.

________________________________________________________________

________________________________________________________________

________________________________________________________________

________________________________________________________________

If you have any suggestions for additional MC experts that we should contact to participate in this manuscript, please provide their names and emails.

________________________________________________________________

________________________________________________________________

________________________________________________________________

________________________________________________________________

In the space below, please include any additional comments.

________________________________________________________________

________________________________________________________________

________________________________________________________________

________________________________________________________________

**Table S3.** Survey given to all microchimerism experts. This survey was filled out online via Qualtrics.
