# Supplementary material for: Identifying Key Questions and Challenges in Microchimerism Biology
Source: Adv Sci (Weinh). 2025 Oct 24;12(48):e14969. doi: 10.1002/advs.202514969 (PMC12752559; doi:10.1002/advs.202514969)
Supplement: Supplementary file 3 — Supplemental Table S3 [file ADVS-12-e14969-s002.docx]

| **Questions** | **Theme 1** | **Sub-theme 1** | **Sub-theme 2** | **MC, MMC, FMC** |
| --- | --- | --- | --- | --- |
| Which animal models are most appropriate for studying the biological functions and disease roles of microchimerism, and for understanding human microchimerism? | Appropriate experimental model systems |  |  |  |
| Is microchimerism defined by the ability to persist long-term and the stem cell nature of the acquired cells? | Definition of microchimerism |  |  |  |
| Does microchimerism also encompass more temporary cells that persist for a while in the maternal body, but that do not engraft, such as fetal erythrocytes or placental trophoblasts that are transferred to the mother during pregnancy? | Definition of microchimerism |  |  | MC |
| Can genetic conflict between microchimeric cells and host cells be reduced in order to improve maternal and fetal outcomes? | Evolution | Adaptation |  | MC |
| Do fetal pregnancy-associated progenitor cells have a competitive advantage over innate maternal stem cells? | Evolution | Adaptation |  | MC |
| What is the adaptive or evolutionary value of microchimerism? | Evolution | Adaptation |  | FMC |
| Which 'evolutionarily paradoxical phenotypes' are caused by microchimerism? | Evolution | Adaptation |  | MC |
| Which animals experience bidirectional microchimerism? Does the type of placenta influence the the transfer of cells? | Evolution | Comparative approach |  | MC |
| How is microchimerism distributed in the animal kingdom in general beyond humans and placental mammals? | Evolution | Comparative approach |  | MC |
| How do microchimeric cells interact with other cells, the host immune system, and with other sources of microchimerism within the same individual? | Function and mechanism | Cell interaction |  | MC |
| What are the mechanisms for recruitment of fetal cells from maternal stem cell niches, once acquired during pregnancy? | Function and mechanism | Cell transmission |  | FMC |
| Is the transfer from host to host active or passive? | Function and mechanism | Cell transmission |  | MC |
| Does the lineage/origin of the microchimeric cells matter for organ specificity? | Function and mechanism | Cell transmission |  | MC |
| What is the role of microchimerism in cardiovascular disease (CVD), particularly atherosclerosis and CVD phenotypes specific to women? | Function and mechanism | Disease vulnerability or protection | Microchimerism in disease | MC |
| How does microchimerism contribute to autoimmune diseases, especially regarding the balance between tolerance and autoimmunity? | Function and mechanism | Disease vulnerability or protection | Microchimerism in disease | MC |
| What is the contribution of microchimerism to cancer development? | Function and mechanism | Disease vulnerability or protection | Microchimerism in disease | MC |
| What is the role of microchimerism in the process of aging? | Function and mechanism | Disease vulnerability or protection | Microchimerism in disease | MC |
| Can fetal cells cause or "cure" disease? | Function and mechanism | Disease vulnerability or protection | Microchimerism in disease | MC |
| What types of maternal injuries or diseases recruit fetal cells? | Function and mechanism | Disease vulnerability or protection | Microchimerism in disease | MMC |
| What is the role of endometrial/uterine microchimeric cells in fertility, decidualization, implantation, placental health, pregnancy health, and subsequent pregnancies? | Function and mechanism | Disease vulnerability or protection | Microchimerism in disease | FMC |
| Can understanding the impact of microchimerism on the maternal and offspring immune systems and *vice versa* help with immune-related disorders, including but not limited to autoimmune disease, fertility issues and even transplantation research? | Function and mechanism | Disease vulnerability or protection | Developmental origins of health and disease | MC |
| Are the associations between microchimerism and later-in-life diseases a reflection of microchimerism serving a functional biological role, or is microchimerism simply a byproduct of pregnancy? | Function and mechanism | Disease vulnerability or protection | Developmental origins of health and disease | MC |
| What are the determinants of maternal microchimerism transfer *in utero* and *post-natally* - specifically, what is the relationship between the maternal immune system and the cells that are transferred? | Function and mechanism | Disease vulnerability or protection | Developmental origins of health and disease | FMC |
| What is the role of microchimerism in development? | Function and mechanism | Disease vulnerability or protection | Developmental origins of health and disease | MC |
| What is the functional role of fetal microchimerism during pregnancy, including impacts on maternal outcomes and immune tolerance? | Function and mechanism | Disease vulnerability or protection | Maternal health and immunity | MMC |
| Does maternal microchimerism shape fetal immune cells towards tolerance only: Or can maternal cells educate fetal T cells towards autoimmunity. Can maternal autoimmunity be transferred to the offspring? | Function and mechanism | Disease vulnerability or protection | Maternal health and immunity | FMC |
| Do microchimeric cells in the maternal brain, and interactions with the immune system, especially in the immediate post-partum time, contribute to challenges in maternal post-partum mental health? | Function and mechanism | Disease vulnerability or protection | Maternal mental health | FMC |
| Does fetal origin microchimerism originating from males and females similarly impact maternal health later in life? | Function and mechanism | Disease vulnerability or protection | Fetal sex and maternal health | MMC |
| How does maternal microchimerism support fetal and neonatal development and immunity? | Function and mechanism | Disease vulnerability or protection | Fetal health and immunity | MMC |
| What is the functional role of maternal microchimerism in infant immunity, both via direct antigen-specific T / B cells or by "educating" the fetal and infant response? Can transferred lymphocytes exert an effect on offspring immunity to infection independently of antigen experience)? | Function and mechanism | Disease vulnerability or protection | Fetal health and immunity | MC |
| Are antigen-specific B cells subject to maternal microchimerism and do they contribute to our antibody repertoire? | Function and mechanism | Disease vulnerability or protection | Fetal health and immunity | MC |
| Does fetal microchimerism play a role in immune tolerance of the fetus during pregnancy? If yes, what is the mechanism? | Function and mechanism | Disease vulnerability or protection | Pregnancy outcomes | MMC |
| What are the mechanisms that allow these foreign cells to persist? | Function and mechanism | Persistence of microchimerism |  | MC |
| Is tolerance against microchimerism actively induced? | Function and mechanism | Persistence of microchimerism |  | MC |
| Why are 'foreign' microchimeric cells not eradicated by the immune system, and which escape mechanisms do they use? | Function and mechanism | Persistence of microchimerism |  | MC |
| How does a woman's microchimerism status fluctuate over her lifetime? | Function and mechanism | Plasticity |  | MC |
| In what way do characteristics of microchimeric cells change in an individual over time? | Function and mechanism | Plasticity |  | MC |
| How does HLA compatibility play a role in quantity and distribution of microchimeric cells in host tissues? | Function and mechanism | Quantity of microchimerism |  | MC |
| What impacts the amount (quantity) of microchimerism transferred transplacentally to the mother during pregnancy (fetal microchimerism) and to the offspring (maternal microchimerism)? | Function and mechanism | Quantity of microchimerism |  | MC |
| Is there a minimum required microchimerism transfer level needed during pregnancy for optimal outcomes? | Function and mechanism | Quantity of microchimerism |  | MC |
| How do small numbers of microchimeric cells impact host biology, and does the impact vary by microchimeric cell type? | Function and mechanism | Quantity of microchimerism |  | MC |
| How does the difference (of whatever kind) of the parental immune systems impact the presence and biology of microchimeric cells? | Function and mechanism | Quantity of microchimerism |  | MC |
| What is the host origin (e.g., bone marrow, peripheral blood, breastmilk) and tissue distribution of microchimeric cells? | Function and mechanism | Tissue dependent microchimerism trafficking |  | MC |
| In what form and in which niche can fetal microchimeric stem cells be found? | Function and mechanism | Tissue dependent microchimerism trafficking |  | FMC |
| Pregnancy-acquired microchimerism can be extended by breast feeding, and milk-associated maternal cells may also enter the neonatal circulation. Does breast-feeding-induced microchimerism affect gut colonization of offspring? How does third-party milk, e.g. provided by milk banks, affect the acquisition of microchimeric cells (non-identical/non-related cells)? | Function and mechanism | Tissue dependent microchimerism trafficking |  | MC |
| Does the function of maternal microchimerism vary according to mechanism of transfer (transplacental vs breastfeeding) or by tissue distribution? | Function and mechanism | Tissue dependent microchimerism trafficking |  | MMC |
| In multiparous mothers, do some pregnancies contribute more microchimerism than others, if so, why? | Mapping the "generational microchimerism" | Intergenerational effect |  | MC |
| What is the impact of maternal microchimerism in human breast milk? | Mapping the "generational microchimerism" | Intergenerational effect |  | MMC |
| Do we see multi-generational transfer of microchimerism - and what is the impact of these different cell lineages? | Mapping the "generational microchimerism" | Transgenerational effect |  | MC |
| What is the frequency of second-order (e.g., siblings) and higher-order (e.g., grandmother) microchimerism? | Mapping the "generational microchimerism" | Transgenerational effect |  | MC |
| Is generational microchimerism another mechanism to explain whether grandparent microchimerism impacts fetal development? If so, how? | Mapping the "generational microchimerism" | Transgenerational effect |  | MC |
| How can we identify the cellular composition and niches of residence within the microchimeric cell populations? | Mapping the "generational microchimerism" | Transgenerational effect |  | MC |
| How can we develop reliable and sensitive technologies to analyze and characterize microchimerism? | Microchimerism detection |  |  | MC |
| How can we determine the individual source of microchimeric cells? | Microchimerism detection |  |  | MC |
| What are the best methods for the unequivocal identification of microchimeric cells? | Microchimerism detection |  |  | MC |
| What is the most efficient and reliable approach to detect and confirm fetal microchimerism, including distinguishing between multiple pregnancies? | Microchimerism detection |  |  | FMC |
| How can we best detect cellular ancestry, and how old is our oldest cell? | Microchimerism detection |  |  | MC |
| How can the therapeutic potential of microchimerism be harnessed (e.g., cell therapy for transplantation or fertility)? What are the long-term health implications of microchimerism from medical interventions? | Microchimerism in interventions, treatment and transplantation |  |  | MC |
| Can we use processes related to fetal-maternal microchimerism to optimize transplant survival rates? | Microchimerism in interventions, treatment and transplantation |  |  | MC |
| Is blood-based detection an adequate marker of microchimerism status in women? | Microchimerism in interventions, treatment and transplantation |  |  | MC |
| Can it be confirmed that pregnancy-acquired microchimerism is akin to a dormant stem cell engraftment? | Microchimerism in interventions, treatment and transplantation |  |  | MC |
| When in the life of the mother or offspring do these dormant cells, such as microchimerism acquired from blood transfusions, become functionally relevant? | Microchimerism in interventions, treatment and transplantation |  |  | MC |
| Does maternal microchimerism contribute to hematopoiesis in the bone marrow? This may shape our lymphoid and myeloid landscapes. | Microchimerism in interventions, treatment and transplantation |  |  | MC |
| Table S3. Full list of edited questions and categorizations. MC = microchimerism. MMC = maternal microchimerism. FMC = fetal microchimerism. Column "MC, MMC, FMC" = determined if the question focused on MC, MMC, or FMC. | | | | |
