# Supplementary material for: Identifying Key Questions and Challenges in Microchimerism Biology
Source: Adv Sci (Weinh). 2025 Oct 24;12(48):e14969. doi: 10.1002/advs.202514969 (PMC12752559; doi:10.1002/advs.202514969)
Supplement: Supplementary file 4 — Supplemental Table S4 [file ADVS-12-e14969-s004.docx]

| **List of Challenges Described by Experts** |
| --- |
| **Complex Diseases** |
| Complexity of Diseases: Autoimmune diseases and other conditions associated with microchimerism are multifactorial, involving interactions between genetics, environment, and immune responses, adding complexity to the research. |
| **Ethical Constraints** |
| Ethical Constraints: Studying microchimerism often involves invasive procedures or long-term monitoring of individuals, which may raise ethical concerns and limit the scope of research. |
| **No Clear Definition of MC** |
| These questions have not been raised, as the MC field is relatively new and so what defines MC has been somewhat fluid as more and more evidence is surfacing. |
| **Diversity of MC** |
| Diversity of Microchimeric Cells: Microchimerism can involve a wide variety of cell types, including fetal, maternal, or donor-derived cells, each potentially playing different roles, making it difficult to generalize findings. |
| We need to expand the breadth of MC research to incorporate all immune cell populations. |
| **Lack of Research Community** |
| Despite numerous studies, the MC field is still small with limited resources, limiting its progress. This is linked to the fact that clear evidence of the importance of MC in health and disease is outlined (and vice versa). |
| Not enough tools to precisely and definitively identify rare cells. |
| The number of researchers is limited. |
| **Limited Interest in Pregnancy, Reproductive Biology, Women's Health** |
| There is a neglect of maternal post-partum mental health that is unfortunate in terms of funding priorities etc. |
| Stem cell researchers, who should appreciate MC, do not generally consider the role or significance of pregnancy and reproductive biology in the development of fetal cell MC. There seems to be more interest and awareness of the role of maternal cell MC in the education of the fetal/neonatal immune system. |
| **Young Field** |
| The MC field is still nascent and much of our understanding focuses on T cells and monocytes. |
| The field is still young. |
| **Lack of Grant Funding** |
| Lack of funding in the field (because the cells are rare, but also because of lack of funding in women's health). |
| Lack of grant funding. This is a multidisciplinary field that lacks an advocacy group in study sections that review grants. There needs to be an increased appreciation of the biological significance of MC. |
| The second largest barrier is funding - it has been exceedingly difficult to get human microchimerism work funded as reviewers would like data demonstrating "function" of the rare maternal cells. Yet, it is not possible to demonstrate function without funding to do so! |
| The field needs to secure funding that may well need to be longer than standard 3 year grants. |
| Funding to date has been limited. |
| **Limitations in Methodology: Lack of Appropriate Animal Model** |
| There was not a real understanding of their normal biological roles as well as in diseases. After decades of research, these major questions remain speculative. In my opinion, mechanistic studies are essential here. Mechanistic studies do not have to use human tissues; a model organism could be ideal. |
| Depletion studies have to make use of antibody or diphtheria toxin methods of depletion. It is difficult to have autoimmunity models that are fully recapitulated in animal models. But these models are becoming available for example for RA and for diabetes. Finally it is possible to do these studies in germ free facilities to avoid confounding factors such as the maternal microbiome. |
| **Limitations in Methodology: Uncontrolled Experimental Designs** |
| We do not have controlled experiments in which we can compare the life histories of organisms with MC with those without MC for positive or negative effects. |
| There is a lack of experimental models that would allow them to be addressed in a functional approach (e.g., by a targeted modulation of microchimeric cells, such as its depletion and modulation). |
| **Limitations in Methodology: Technology** |
| Methodological challenges - like measuring how single cells or clusters of cells are interacting with their immediate environments - is a big barrier. |
| There is an over-reliance on FISH studies to demonstrate MC. There is a need for more modern technologies that can validate rare events such as fetal cell MC. |
| We do not have solid evidence for the pervasiveness of the phenomenon within or across taxa due to technical limitations for easily measuring it. |
| We do not know how MC cells escape immune surveillance, and how MC tolerance is maintained or lost. Animal models to test mechanisms of immune tolerance are restricted by the number of available trackable model antigens that could represent the full microchiome. Human studies are confounded by the fact that the majority of MC cells may be tissue-resident (tissue sample required). Detection methods with high sensitivity and specificity (PCR-based) preclude the interrogation of their functional attributes. Cancer or autoimmune-diseased tissue may only be available with fully-established disease – precluding to investigate disease development. |
| Not enough people realize the existence of microchimerism, much less study these cells. |
| The unambiguous detection of MC is cumbersome and a technical challenge because detection must be confirmed by a second independent method. Images can be adapted (histogram) to suggest a positive staining for a marker indicating MC. GFP, an often used marker, is inappropriate due to many tissues or intra- and extracellular components exhibit quite some autofluorescence in the FITC channel. Data reported by qPCR or ddPCR come at low frequencies and, moreover, do not allow spatial analysis. |
| The largest barrier to answering these questions is related to technology development to be able to study the function of very rare maternal cells. Prior work has largely focused on the use of discordant DNA markers, which do not allow for functional assessment of the transferred cells. In addition, the current tools we have worked to develop (single cell RNAseq, discordant HLA antibodies, XIST) all have limitations, are difficult to implement, are expensive, and are not largely scalable (e.g. a different marker has to be used for each maternal-infant pair). |
| **Limitations in Methodology: Limited Longitudinal Data and Challenging Data Collection** |
| Methods for detecting microchimerism are challenging. The fact that these are rare cells has led to skepticism in the field of microchimerism research. Critics are skeptical that these projects are worthy of funding mechanisms and want evidence there is a functional impact on the host biology. Further, methods for detecting microchimerism are challenging, expensive and very time consuming. Because microchimerism research lacks funding, many microchimeric studies are using biobank samples that were not collected for the purpose of tracking microchimerism. More research programs need to be developed to include complete pregnancy histories as well as sampling at multiple time points during pregnancy and the postpartum period, and extensive sampling of family members. Lastly, the methodology is challenging and time consuming - a better integrated community of microchimerism researchers - that can share protocols and research pipelines will facilitate better microchimerism research! |
| Studying MC mechanistics in the human placenta in vivo is ethically and logistically challenging during pregnancy, though ex vivo studies are showing potential for beginning to address such questions. |
| Longitudinal biobanks from pregnancy into older maternal age are rare, and therefore associations between fetal MC in pregnancies with adverse outcomes of placental etiology (e.g. preeclampsia, preterm premature delivery, fetal growth restriction) and maternal cardiovascular lesions are challenging to study in humans. |
| Limited Longitudinal Data: Comprehensive long-term studies following individuals with microchimerism are limited, making it challenging to assess the impact of microchimerism over a person's lifetime. |
| To assess the microchiome on a population level, considering all transgenerational layers of MC, non-shared HLA-haplotypes and deletion/insertion polymorphisms between all individuals need to be determined. Generating an individual’s microchiome profile, blood samples (the most clinically accessible sample) from all family members are required. Decades after pregnancy/gestation, MC may only be present within tissues (tissue sample required). |
| While endometrial biopsies from healthy uteri to characterize the physiological microchimeric state are not readily available (except potentially via menstrual blood), biopsies from women with infertility are routinely taken. However, the non-shared HLA-haplotype or other polymorphisms need to be determined and all other contributing sources of MC should be considered, which may not be feasible in the case of (e.g., recurrent pregnancy loss. The requirements for a robust and impactful study are complex.) |
| Whether their effect is indirect (fetal cell instruction) or direct (e.g., maternal pathogen-specific cells target infection) remains to be demonstrated in humans and preclinical models to ultimately mimic functions in therapeutic settings. |
| As epidemiologists we need relatively big datasets, and thus affordable and convenient testing. Y-chromosome detection is cheap and easy. |
| It is not easy to prospectively follow a large group of women over long periods of time with consecutive blood and tissue sampling. |
| MC cells may home to compartments (bone marrow, lymph nodes) that are generally challenging to collect. |
| This is a challenging field to work in yet more researchers need to be drawn in. Modelling requires complex, long term and expensive experiments fraught with being disrupted by (for example) maternal rejection of offspring. Human studies also need to be long term. |
| MC is challenging to study both in relation to adequate methodologies and relevant experimental systems. The first issue is currently addressed with the development of several technologies that now can help address the fundamental questions in the MC field. |
| **Limitations in Methodology: MC is Rare and Hard to Detect** |
| I think the main reasons for not being answered are that MCs are hard to study (technically some challenges), that the amount of cells isn't that high, the associations aren't always very strong, but also that the field doesn't always get the appreciation it deserves. Hopefully with this new consortium and also with all new technical possibilities there will be a big boost! |
| Rare cells are hard to study. |
| Microchimeric cells are rare and not easy to collect and analyze. It has been simpler to look for single markers (Y chromosomes, non-inherited MHC) than perform a multi-locus identification of actual individuals. |
| Fetal cells are very rare in the mother, and can only most easily be obtained from the peripheral circulation. To study fetal cells in vivo, animal models must be used. They are expensive and limit experiments to facilities that can breed mice. |
| Technical Challenges: Microchimeric cells are often present in very low numbers, making their detection and characterization challenging, especially within host tissues. |
| The major challenge I see is the rarity of such cells and consequently the ability to study them ex vivo and in tissue context. |
| These rare cells are difficult to detect (signal-to-noise ratio), isolate (tools to target unique markers are underperforming (poor antibody-specificity), and enrich (in vitro expansion may introduce artifacts). Mechanisms of beneficial effects remain unknown – no blueprint yet to re-create a function. A major open question is how the stemness of a microchimeric cell is different from a metastatic tumor cell or a tissue-resident stem cell. |
| For the identification of MCs, there lacks a reliable technique that can easily detect MCs in tissues / biological specimens. Detection usually requires prior information of the maternal / fetal genotype, which limits the use of most tissues in tissue banks. |
| I think most questions remain un/under-studied because we lack sufficient assays for microchimerism. Further, assaying tissues is challenging, but new genomics approaches may make this more feasible (e.g., has anyone looked for microchimeric cells using single cell approaches?). |
| Very low percentage of these makes it difficult to enrich them in a proper way. Follow-up of MC cells over time would mean necessity of sequential material from the same individual, which often is difficult. |
| A significant hurdle is the low sensitivity of detection, since microchimeric cells are generally ‘one in a million’, depending on the organ. With such frequencies, detection methods such as flow cytometry quickly reach their limits. Single cell-based sequencing methods, on the other hand, require a minimum amount of genetic material, which is also very difficult to isolate reliably from such small numbers of cells. However, enrichment of microchimeric cells, e.g., by magnetic-activated cell sorting, prior to flow-cytometry or single cells analyses have shown that these limitations can be significantly overcome and advances understanding of the functional role. |
| It is hard to measure what impact genetic variants of molecules have on their biology. Especially in case of the MHC genes/alleles because 1) there are several thousand different variants and 2) they are basic to the immune system which is complex in itself. |
| Main reasons for the lack of data on the impact of rare cells I think are due to tricky analysis. 1) Monitoring of the impact of a few cells needs very specific ideas of what to look at. 2) Few cells generate probably only a very local impact that is diluted below detection threshold in bulk analysis. 3) The less cells to be interrogated the better characterized the cells need to be to anticipate their impact that, in turn, needs to be analyzed in a very specific analysis/high resolution. |
| Table S4. Expert feedback describing the major challenges to microchimerism research. MC = microchimerism. |
